# Astragaloside–Brucea Javanica Oil Nanoemulsion Regulates Glycolysis in Oral Squamous Cell Carcinoma Through AURKA-Mediated PI3K/AKT/HIF-1α Pathway

**DOI:** 10.3390/ph18121783

**Published:** 2025-11-24

**Authors:** Runqiang Liu, Juan Zhan, Yihan Lai, Yujie Ma, Wei Wang, Lin Jiang, Yisen Shao

**Affiliations:** 1 Clinical Medical College of Jiangxi University of Chinese Medicine, Nanchang 330006, China; jegengxss@email.ncu.edu.cn (R.L.); zhanjuan@jxutcm.edu.cn (J.Z.); laiyihan@jxutcm.edu.cn (Y.L.); wangwei42@jxutcm.edu.cn (W.W.); jianglin1@jxutcm.edu.cn (L.J.); 2 School of Stomatology, Nanchang University, Nanchang 330006, China; mayujie@email.ncu.edu.cn

**Keywords:** network pharmacology, bioinformatics analysis, OSCC, AURKA, astragaloside IV, brucea javanica oil

## Abstract

**Background**: Oral squamous cell carcinoma (OSCC) is a common malignant tumor of the head and neck, and glycolysis plays a key role in its development. In the early stages of the study, we prepared a nanoemulsion containing Astragaloside IV (AS-IV) and Brucea javanica oil (BJO). This Astragaloside–Brucea Javanica Oil nanoemulsion (AS/BJO-NE) demonstrated a stronger inhibitory effect on the proliferation, invasion, and migration of OSCC cells than either AS-IV or BJO alone. Preliminary experiments also showed that AS/BJO-NEs inhibited glycolysis in OSCC cells. The aim of this study was to investigate how AS/BJO-NEs act against OSCC by targeting glycolysis-related genes and pathways. **Methods**: Prepare AS/BJO-NEs and determine their particle size, PDI, and potential. Network pharmacology and bioinformatics analysis were employed to identify the core genes and pathways of AS/BJO-NEs involved in regulating glycolysis in OSCC. In vitro and vivo experiments were performed to investigate the effects of AS/BJO-NEs on OSCC tumor development and core gene expression levels. **Results**: Aurora kinase A (AURKA) is a critical target through which AS/BJO-NEs regulate glycolytic metabolism in OSCC. Combined in vitro and in vivo experiments revealed that AS/BJO-NEs suppress glycolysis-related enzymes HK2 and PKM2 through the AURKA/PI3K/AKT/HIF-1α signaling axis, consequently inhibiting OSCC proliferation, invasion, metastasis, and subcutaneous tumorigenesis. **Conclusions**: Bioinformatics analysis combined with in vitro and vivo experiments demonstrated that AS/BJO-NEs downregulate OSCC glycolysis via the AURKA/PI3K/AKT/HIF-1α pathway at the metabolic level, thereby inhibiting OSCC progression. Elucidation of this mechanism provides theoretical support and experimental evidence for the anti-OSCC effects of AS/BJO-NEs.

## 1. Introduction

Oral squamous cell carcinoma (OSCC) is a common malignant tumor of the oral cavity. It accounts for 2–4% of all cancers [1] and is characterized by its aggressiveness and tendency to recur locally and to metastasize distantly [2,3]. The development of OSCC is complex. Recent studies have revealed that it involves processes like the Warburg effect and epithelial–mesenchymal transition (EMT) [4]. Glycolytic metabolism plays a pivotal role in the development of OSCC. The glycolytic flux of OSCC malignant cells has been shown to be 3–5 times higher than that of normal epithelial cells, thus demonstrating a significant Warburg effect. Simultaneously, lactic acid produced by glycolysis leads to extracellular microenvironment acidification, thereby promoting cancer cell invasion and metastasis [5]. The management of OSCC is currently approached through a multidisciplinary lens, encompassing surgical interventions, radiotherapy, and chemotherapy. While this has improved the survival rate of OSCC patients to a certain extent, the side effects of the treatment have seriously impacted their quality of life [6]. Traditional Chinese Medicine (TCM) has gained widespread attention due to its advantages, including multi-component and multi-target actions, and minimal side effects [7]. It has been demonstrated that TCM exerts anti-tumor effects by regulating aerobic glycolysis and hindering angiogenesis and EMT [8,9].

Astragaloside-IV (AS-IV), a bioactive constituent of *astragalus* an inhibit tumor cell proliferation and promote tumor cell apoptosis in tumor therapy, and is widely used [10,11]. Studies demonstrate that AS-IV suppresses viability and glycolysis in hepatocellular carcinoma cells (HCCs) [12]. Brucea javanica oil (BJO) demonstrates precise anti-tumor efficacy and is widely used in treating various cancers [13,14]. Our team discovered that BJO inhibits the progression of tongue squamous carcinoma cells by downregulating aerobic glycolysis, thus demonstrating anti-tumor efficacy against this malignancy [15,16].

As a novel drug delivery system, Chinese medicine nano-preparations have garnered widespread attention [17,18]. Owing to their unique physicochemical properties, high permeability, and prolonged retention effects, these preparations can enhance drug efficacy against tumor cells while reducing toxic side effects on normal tissues [19]. BJO has been proven to prolong circulation time and enhance bioavailability when utilized as a nanomedicine carrier [20]. Therefore, based on the anti-tumor properties of AS-IV and BJO, as well as the advantages of TCM nano-formulations, our team created the Astragaloside–Brucea Javanica Oil nanoemulsion (AS/BJO-NE) by combining BJO with AS-IV using nanotechnology. This work has been granted a national patent (Patent Number: ZL2024 1 0606132.2).

Network pharmacology is regarded as a new field in drug discovery and the latest frontier of systems drug research [21]. Machine learning technology is a significant aid in multi-target drug discovery and is increasingly commonly used to predict the impact of related characteristic indicators on patient survival. The construction of accurate prognostic assessment models via machine learning facilitates the identification of effective biomarkers and therapeutic targets. This approach ultimately enables precise treatment strategies for patients grounded in molecular mechanisms [22,23]. Furthermore, the integration of machine learning with multi-omics research like transcriptomics and single-cell transcriptomics has further advanced multiple fields in medicine [24,25,26].

In this study, network pharmacology and bioinformatics analysis combined with in vitro and in vivo experiments demonstrated that AS/BJO-NEs downregulate OSCC glycolysis via the AURKA/PI3K/AKT/HIF-1α pathway, thereby inhibiting OSCC proliferation, invasion, metastasis, and subcutaneous tumorigenesis. Elucidation of this mechanism provides theoretical support and experimental evidence for the anti-OSCC effects of AS/BJO-NEs.

## 2. Results

### 2.1. Network Pharmacological Analysis of AS/BJO-NEs Against OSCC

#### 2.1.1. Acquisition of Target Sites for AS/BJO-NEs in the Treatment of OSCC

We obtained a total of 20 active ingredients and 228 target genes of AS/BJO-NEs. OSCC-related targets were obtained from five disease databases (GeneCards, DisGeNET, TTD, PharmGKB, and OMIM). After processing with UniProt to remove blank entries and duplicates, a total of 5778 target genes were retained (Supplementary Table S1).

#### 2.1.2. Construction and Enrichment Analysis of PPI Network

Venn analysis obtained 172 intersecting target genes of AS/BJO-NEs against OSCC (Figure S1A). A visualization of drug-intersecting target disease network maps was created with Cytoscape software (version 3.10.2) (Figure S1C). The 172 intersecting targets were entered into the STRING database to analyze their interactions. A minimum interaction score of >0.4 was set to identify the stronger associated proteins and a PPI network was generated using Cytoscape (Figure S1B). In addition, the “ClusterProfiler” (version 4.12.0) and “ggplot2” (version 3.5.1) R packages were used to analyze and visualize KEGG and GO enrichment for 172 common targets. KEGG is mainly enriched in PI3K-Akt signaling pathway. In GO, it is mainly enriched in glucose homeostasis, mitochondrial transport, and other biological processes (Figure S1D,E). This suggests that the PI3K-Akt signaling pathway may be the main pathway through which AS/BJO-NEs exert their effects, and the influence on glucose homeostasis and mitochondrial transport might be the main mechanism by which AS/BJO-NEs act.

### 2.2. OSCC Machine Learning Prognostic Risk Model for Glycolysis and Consistency Clustering Analysis

#### 2.2.1. Construct and Screen a Prognostic Risk Model for OSCC Glycolysis-Related Genes

**Figure 1:**
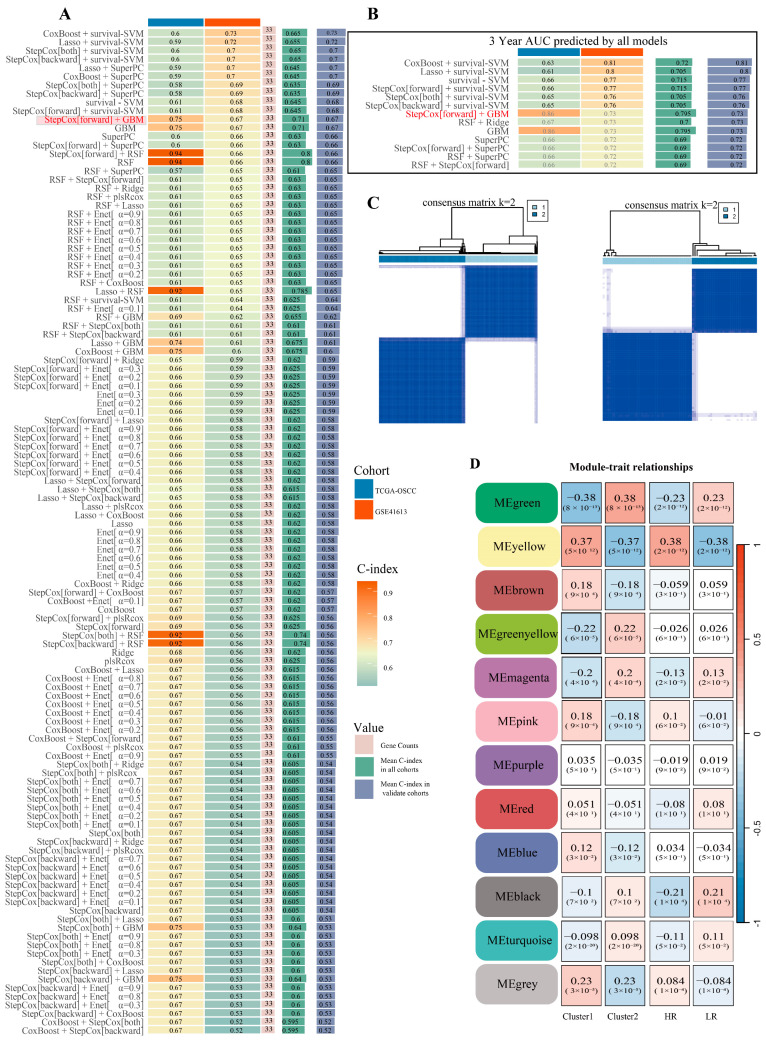
Construction of a machine learning prognostic risk model and WGCNA: (**A**) C-index numerical visualization of a machine learning prognostic risk model for OSCC glycolysis-related genes constructed by combining multiple algorithms. (**B**) Visualization of the 3-year survival AUC portion of the machine learning prognostic risk model. (**C**) The TCGA-OSCC training set and the GSE41613 validation set of glycolysis model gene risk subgroups were obtained by clustering analysis, respectively. (**D**) Heatmap showing correlation between gene modules and selected phenotypes.

First, univariate Cox analysis identified 45 glycolysis-related genes significantly associated with poor prognosis in OSCC patients. These were selected as characteristic genes (Supplementary Table S2). Then, using the transcriptome data and clinical survival data of TCGA-OSCC and GSE41613 as the training set and validation set of the machine learning model, respectively, we constructed an OSCC glycolysis-related gene prognosis risk model including 117 algorithm results through R 4.4.1 packages (Figure 1A).The 3-year survival AUC results of patients under various algorithm combinations were visualized, with partial results shown in Figure 1B. Considering the C-index and 3-year AUC of the TCGA training set and the GEO validation set comprehensively, Step Cox[forward] + GBM was selected as the optimal algorithm, which resulted in the optimal OSCC glycolysis-related gene prognostic risk model, including a total of 33 risk model genes. Step Cox [forward] + GBM achieved C-index values of 0.75 and 0.67 on the training and validation sets, respectively, with 3-year AUC values of 0.86 and 0.73. The specific model results are presented in Supplementary Table S3. We visualized the K-M survival analysis of the risk model in the two datasets and found that the survival rate of high-risk patients was significantly lower than that of the low-risk group (Figure S2A, *p* < 0.01), indicating that the risk model had excellent predictive performance for adverse prognosis.

#### 2.2.2. Consistency Clustering Analysis and External Verification Based on the Optimal Model Genes

Furthermore, using the selected model genes, we performed unsupervised cluster analysis on the samples included in the TCGA training set and the GEO validation set using the “ConsensusClusterPlus” R package (version 1.68.0) to classify the samples into different glycolytic subclasses, and the clustering results were optimal at K = 2 (Figure 1C). Subsequently, we performed Kaplan–Meier survival analyses between the subgroups (C1 and C2) of the two datasets separately, and observed a significant difference between them (Figure S2B, *p* < 0.001). The results indicated that the first subgroup had a poorer survival prognosis, defining C1 as the poor glycolytic prognostic subtype. KEGG enrichment analysis was performed on the differential genes that satisfied Log2FC > 0.5 and *p* < 0.5 (Supplementary Table S4) after difference analysis between the subclasses of the two datasets. The results revealed that the differentially expressed genes were primarily enriched in the PI3K-AKT, HIF-1α, and TNF-α pathways (Figure S2C,D). The oncogenic signaling pathways were significantly enriched in group C1, which may explain its poorer prognosis. This result also indirectly validates the outcome of machine learning. TIDE analysis showed higher TIDE scores for subtype C1 in both datasets (Figure S2E), which is consistent with subtype C1 having a worse survival prognosis. GSVA immune infiltration analysis of both datasets showed that C1 significantly influenced the infiltration of immune cells, and we present them as the average expression of each sample for immune cells (Figure S2F,G). Box plots are shown in “Figure S2H,I”. Pointwise analysis of immune checkpoints of the two datasets showed that the C1 subtypes had a significant increase in CD276, CD48, CD28, CD244, and TNFRSF14 showed downregulated results (Figure S2J,K). The results indicate that genes identified by the machine learning-based glycolysis risk model substantially affect tumor immune microenvironment dynamics and patient survival prognosis, and may affect tumor glycolysis through multiple pathways to influence tumor progression and consequently patient survival prognosis.

Not only that, we also conducted two additional transcriptome data analysis (GSE65858 and GSE85446) for external validation to further prove the reliability of a set of genes obtained from the construction of the prognostic model. Based on the model genes, we scored the aforementioned two gene samples through lasso-Cox analysis (the risk score = total risk score = Σ (expression level of gene × LASSO-Cox coefficient of gene)). After obtaining the risk score, we distinguished the high-risk group from the low-risk group. Finally, we found that the high-risk score in GSE65858 and GSE85446 had a significant impact on the poor prognosis of patients. Multivariate regression analysis indicated that the risk score was not affected by confounding factors (Figure S3, GSE65858: Survival analysis, *p* < 0.001; multivariate regression analysis, *p* < 0.001, HR = 12.02; GSE85446: Survival analysis, *p* < 0.001; multivariate regression analysis, *p* = 0.008, HR = 2.88).

Finally, Venn analysis found that AS/BJO-NEs acted on four of the model genes, including AURKA, CDK1, ADORA2B, and G6PD (Figure S2L). These are the glycolytic hub genes (GHGs) of the AS/BJO-NEs against OSCC.

### 2.3. WGCNA Identified the Core Targets Related to Glycolysis That Are Targeted by AS/BJO-NEs

#### 2.3.1. WGCNA Identifies the Modules Associated with High Risk and Poor Prognosis

This part aimed to identify genes associated with both the poor-prognosis glycolysis subtypes (C1) and the OSCC glycolysis high-risk model group as glycolysis-related genes (GRGs) for inclusion in the screening of targets for regulation of OSCC glycolysis by AS/BJO-NEs. The WGCNA algorithm implemented in R analyzed TCGA-OSCC differential genes (Supplementary Table S5), and chose a soft threshold of β = 9 (Figure S4B) with a minimum module size of 50 to obtain 12 modules (Figure 1D), of which the yellow modules have higher correlation. We visualized the genes of the yellow modules (Figure S4C). To improve the credibility of the results, we included more than 5000 genes with large gene variations in the GSE41613 dataset by using the R language VAR function and repeated the above clustering operation to obtain a total of 13 modules (Figure S4D–J), where the yellow and red modules have higher correlations. Finally, Venn analysis of modular genes from WGCNA and the target genes of AS/BJO-NEs against OSCC identified nine glycolysis-related genes (GRGs) (Figure S4K).

#### 2.3.2. Analysis of Poor Prognosis of GHGs and GRGs and Correlation Analysis with Glycolysis

Univariate Cox regression (Figure S4L) and K-M survival analysis (Figure S5A–K) were conducted on the aforementioned eleven genes (GHGs and GRGs). The results demonstrated that AURKA and PLK1 significantly impacted patient survival and showed higher hazard ratios (AURKA: HR = 1.291, *p* = 0.02; PLK1: HR = 1.354, *p* = 0.008). The further correlation analysis of a total of eleven genes with glycolysis, and AURKA and MET had a higher significant correlation with OSCC metabolic levels (Figure S6A–K, AURKA: R = 0.47, *p* < 0.001; MET: 0.47, *p* < 0.001). In addition, ROC analysis of the TCGA-OSCC and GSE30784 datasets (Figure S5L and Figure S6L, respectively) demonstrates that AURKA outperforms other genes in distinguishing OSCC from non-OSCC samples (TCGA-OSCC, AURKA, ROC = 0.967; GSE30784, AURKA, ROC = 0.862). Additionally, our differential analysis revealed that AURKA exhibited the most significant differential expression in OSCC (Supplementary Table S5, log2FC of AURKA = 1.91).

In summary, we identified AURKA as a core target gene for AS/BJO-NEs to influence OSCC development through the glycolytic pathway.

### 2.4. Bioinformatics Analysis of AURKA

**Figure 2:**
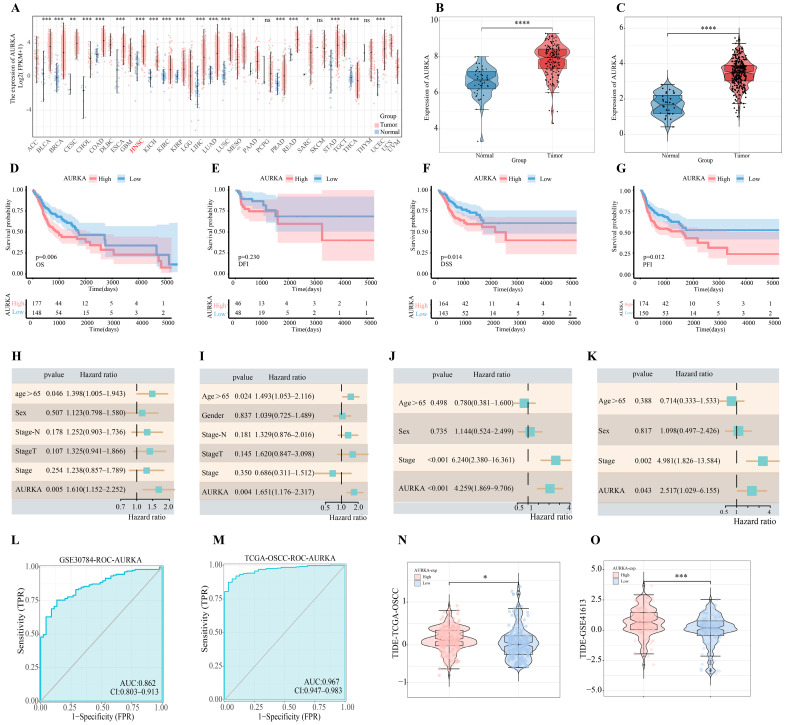
AURKA was significantly associated with OSCC survival prognosis, immune escape and altered tumor microenvironment: (**A**) Analysis based on TCGA-Pan-Cancer transcriptome data revealed that AURKA is highly expressed in a wide range of tumors. (**B**,**C**) There was a significant difference in AURKA expression between OSCC samples and normal controls in TCGA-OSCC and GSE30784. (**D**–**G**) We analyzed the effect of AURKA expression on survival outcomes such as OS, DFI, DSS, and PFI in TCGA-OSCC samples. (**H**,**I**) Univariate (**H**) and multivariate COX analyses (**I**) were performed on AURKA in TCGA-OSCC. (**J**,**K**) Univariate (**J**) and multivariate COX analyses (**K**) were performed on AURKA in GSE41613. (**L**) Visualization plot of AURKA’s ROC analysis on GSE30784 transcriptome data. (**M**) Visualization plot of AURKA’s ROC analysis on TCGA-OSCC transcriptome data. (**N**,**O**) TIDE analysis of each sample from two different datasets. “ns” is not significant, * *p* < 0.05, ** *p* < 0.01, *** *p* < 0.001, **** *p* < 0.0001.

Analysis of the TCGA-Pan-Cancer dataset revealed elevated AURKA expression across multiple tumor types relative to normal tissues (Figure 2A). Furthermore, analysis of the OSCC datasets revealed consistently significant differences in AURKA expression between OSCC samples and normal controls (Figure 2B,C).

In survival analysis of TCGA-OSCC, we extracted Overall Survival (OS), Disease-Free Interval (DFI), Disease-Specific Survival (DSS), and Progression-Free Interval (PFI) data (Figure 2D–G). The optimal AURKA cutoff value was determined for two datasets, TCGA-OSCC and GSE41613 (Figure S7A–E), enabling stratification into high- and low-expression groups. Subsequently, univariate and multivariate Cox analyses were performed, incorporating confounders such as age, clinical grade, etc. The results are presented in Figure 2H–K. All these results indicate that the high expression of AURKA significantly affects the survival prognosis of OSCC patients.

Figure 2L,M show the accuracy of AURKA in predicting the cancer status of samples, with an area under the curve (AUC) > 0.85, indicating that AURKA has good accuracy in differentiating OSCC samples from normal controls.

TIDE analysis showed that OSCC patients had higher TIDE scores when AURKA was highly expressed (Figure 2N–O), suggesting that OSCC tumor cells are prone to immune escape and less likely to benefit from immunotherapy in the presence of high AURKA expression. Moreover, the immune infiltration analysis of both datasets revealed that elevated AURKA expression suppressed immature dendritic cells (IDCs), mast cells, and T follicular helper (Tfh) cells while enhancing activated CD4 T cells and type 2 T helper (Th2) cells (Results from TCGA-OSCC and GSE30784 shown in Figure S7F,H, respectively). Immune checkpoint correlation analysis of both datasets showed that high AURKA expression promoted CD276, CD44, HHLA2, and LAG3 expression (with analysis of TCGA-OSCC and GSE30784 shown in Figure S7G,I, respectively).

Furthermore, drug sensitivity analysis demonstrated that AURKA overexpression confers resistance to therapeutic agents including 5-fluorouracil, foretinib, and apicidin (Figure S7J).

### 2.5. Analysis of AURKA-Related Pathways and Their Correlation with Glycolysis

#### 2.5.1. Transcriptomic Analysis of Enrichment of AURKA-Related Pathway

**Figure 3:**
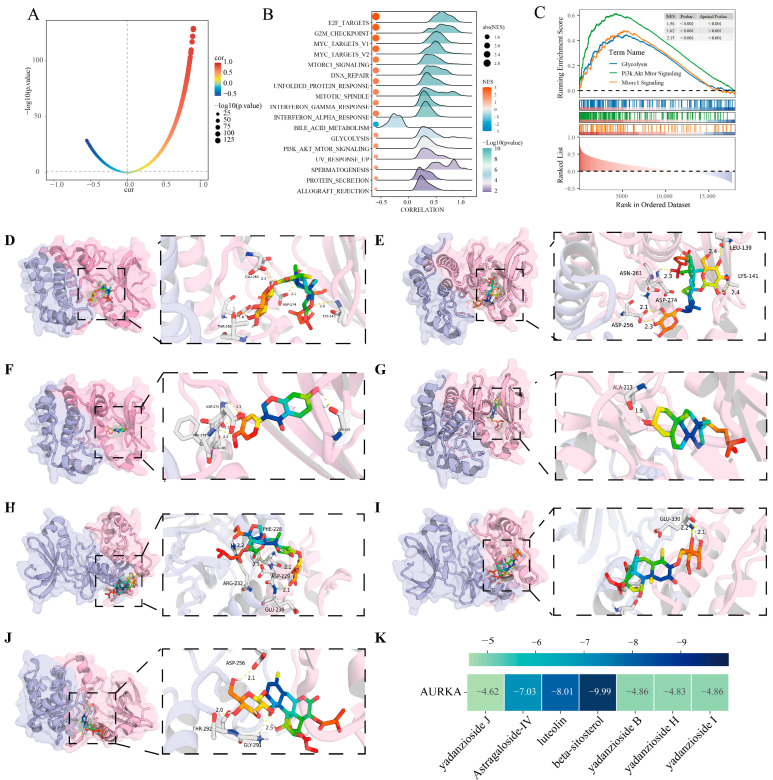
GSEA and molecular docking results of AURKA: (**A**) Correlations between AURKA and other genes in the TCGA-OSCC transcriptome data were calculated and visualized. (**B**) Visualization of the mountain range diagram of the results of the GSEA analysis. (**C**) AURKA was found to be positively correlated with PI3K/AKT/MOTR pathway activity and glycolytic metabolism levels. (**D**) Docking visualization of AURKA with the yadanzioside J component from BJO. (**E**) Docking visualization of AURKA with the AS-IV. (**F**) Docking visualization of AURKA with the luteolin component from BJO. (**G**) Docking visualization of AURKA with the beta-sitosterol component from BJO. (**H**) Docking visualization of AURKA with the yadanzioside B component from BJO. (**I**) Docking visualization of AURKA with the yadanzioside H component from BJO. (**J**) Docking visualization of AURKA with the yadanzioside I component from BJO. (**K**) Docking binding energy thermogram of AURKA with the active components of AS/BJO-NEs.

We conducted GSEA analysis using TCGA-OSCC and GSE30784. AURKA correlation with other genes was calculated and ranked (Supplementary Table S6), and the correlation visualization was shown as Figure 3A. GSEA enrichment analysis was conducted using the predefined gene set “h.all.v2024.1.Hs.symbols.gmt”. The results further showed that AURKA expression was indeed positively correlated with glycolytic metabolism (Figure 3B,C and Figure S8A,B), and not only that, AURKA expression also showed a positive correlation with the activation of the PI3K pathway, a classical pathway associated with glycolysis (Figure 3C and Figure S8C). The GSVA results further revealed elevated glycolytic metabolism and increased PI3K pathway activation in samples with high AURKA expression (Figure S8D–G). And this result could also be reflected at the single-cell level.

#### 2.5.2. Single-Cell Transcriptomics Validate the AURKA-Related Pathways

First, we obtained and visualized the annotation information of the GSE188737 single-cell transcriptome dataset using the “seurat” R package (version 5.2.1) (Figure S8H), and found that AURKA was mainly expressed in epithelial cells (Figure S8I). We performed dimensionality reduction clustering on epithelial cells (Figure S8K) and scored them based on the glycolysis pathway gene set and the PI3K/AKT pathway gene set (Supplementary Table S2) using the “AUCell” R package (version 1.20.0). When AURKA was highly expressed, glycolysis levels were higher in both primary tumor foci and lymph node metastatic foci, and PI3K/AKT pathway activation levels were also higher (Figure S8L–O). The “scMETABOLISM” R package further illustrates the higher glucose uptake and glycolytic metabolism of epithelial cells when AURKA is highly expressed (Figure S8P–S).

This further supports the results of the previous transcriptome data analysis that AS/BJO-NEs may affect tumor development by decreasing the expression of AURKA and then downregulating the glycolysis level of OSCC through the PI3K/AKT pathway.

### 2.6. Molecular Docking

The results revealed that the binding energy of the AS-IV-AURKA complex was −7.03 kJ/mol, the binding energy of the luteolin–AURKA complex was −8.01 kJ/mol, while that of the beta-sitosterol–AURKA complex was −9.99 kJ/mol. These molecule ligands exhibited favorable binding stability with AURKA, as their binding energies were all lower than −5 kJ/mol. PyMOL2 was then used to visualize the docking results (Figure 3D–J), and the R package “pheatmap” (version 1.0.12) was used to visualize the resulting data and present the heatmap (Figure 3K). The results indicate that the active components of AS/BJO-NEs likely inhibit OSCC by binding tightly to AURKA.

### 2.7. Composition and Preparation of AS/BJO-NEs

The AS/BJO-NEs were prepared by using the high-pressure homogenization method (Highpressurehomogenisation, HPH). The results obtained from the Malvern laser particle size analyzer indicated that the particle size of AS/BJO-NEs was 239.033 ± 5.907 nm, the PDI value was 0.161 ± 0.283, and the potential was 0.679 ± 0.304 mV. The particle size of the nanoemulsion was approximately 200 nm, which met the requirements. In addition, we had previously verified through the CCK8 tumor inhibition experiment the influence of the ratio of AS-IV to BJO components on the anti-OSCC effect of AS/BJO-NEs, and excluded the effects of auxiliary components such as soybean phospholipids and glycerol on OSCC (Supplementary Table S7). Finally, the UPLC/MS spectrum indicated that the preparation of AS/BJO-NEs did not change the main components used (Figure S9). AS/BJO-NEs demonstrate superiority due to their sustained-release effect, and exhibit stronger tumor inhibitory effects than AS-IV and BJO single-component drugs. More characterization information such as particle size and stability can be found by referring to the patent number (ZL2024 1 0606132.2) or the patent application number (CN202410606132.2).

### 2.8. AS/BJO-NEs Inhibit OSCC Proliferation Invasion Metastasis and Glycolytic Metabolism

**Figure 4:**
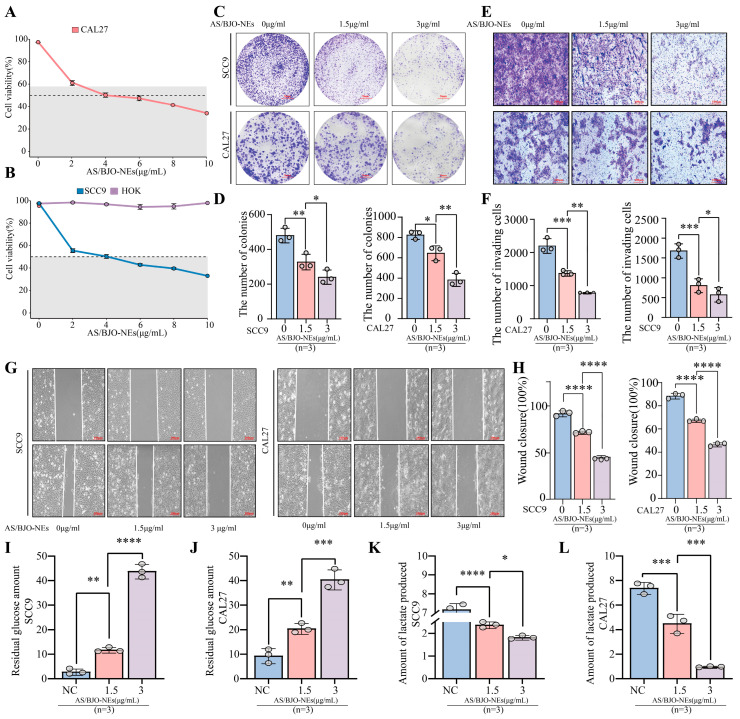
AS/BJO-NEs inhibit OSCC cell proliferation migration invasion and glycolytic metabolism: (**A**,**B**) CCK8 detects the effect of AS/BJO-NEs at different concentrations on the viability of SCC9, HOK, and CAL27 cells. The dotted line indicates the 50% mark. (**C**) Colony formation assay of SCC9 and CAL27. (**D**) The results of the transwell invasion assay showed that the degree of inhibition of OSCC invasion by AS/BJO-NEs increased with concentration and the results were significant. (**E**,**F**) Cell scratch test to determine the effect of different concentrations of AS/BJO-NEs on OSCC migration levels. (**G**,**H**) AS/BJO-NEs inhibited cell migration. (**I**–**L**) AS/BJO-NEs suppressed glucose uptake and lactate production of OSCC cell. * *p* < 0.05, ** *p* < 0.01, *** *p* < 0.001, **** *p* < 0.0001. The circles on the bar chart represent the sample size or the number of repetitions, while the bars represent the mean ± SD. *n* = 3 indicates three repetitions for each group.

The CCK-8 assays revealed dose-dependent suppression of OSCC cell viability (SCC9, CAL27) by AS/BJO-NEs, while HOK cell proliferation remained unaffected after 24 h treatment versus controls (Figure 4A,B). The IC50 values of CAL27 cells and SCC9 cells were 3.346 μg/mL and 3.403 μg/mL. Additionally, we established a blank control group, an empty nanoemulsion group (without AS-IV and BJO), and an AS/BJO-NE group (IC50 concentration) for the CCK-8 assay, thereby ruling out the influence of the empty nanoemulsion on the OSCC cell line (Figure S10).

Based on these results, we selected AS/BJO-NEs 1.5 μg/mL as the low-concentration group and 3 μg/mL as the high-concentration group for the subsequent experiments. We then used colony formation assay to confirm the inhibitory effect of AS/BJO-NEs on cell proliferation (Figure 4C,D). After 24 h treatment with AS/BJO-NEs, colony formation assays revealed significantly fewer clones compared to controls. These findings aligned with CCK-8 assay results, demonstrating that AS/BJO-NEs effectively suppressed the proliferative capacity of OSCC.

Furthermore, the other experimental results demonstrated that AS/BJO-NEs treatment reduced the number of OSCC cells penetrating the Matrigel basement membrane (Figure 4E,F) and significantly inhibited cell migration (Figure 4G,H). Concurrently, glucose uptake and lactate production by OSCC cells were decreased (Figure 4I–L), indicating that AS/BJO-NEs suppressed OSCC cell invasion, migration, and glycolytic metabolism. Notably, the inhibitory effects were significantly stronger in the high-concentration group than in the low-concentration group.

The in vivo experiments of establishing subcutaneous tumor xenografts using wild-type SCC9 cells also demonstrated that, compared with the control group (0.9% saline solution group), the high-concentration group (100 mg/kg) and the low-concentration group (50 mg/kg) of AS/BJO-NEs could significantly inhibit the proliferation of tumor cells (Figure S11A–C), and the effect of the high-concentration group was stronger than that of the low-concentration group. Specifically, compared to the control, the tumor volume reduction rates were 41% in the low-concentration group and 68% in the high-concentration group.

### 2.9. AS/BJO-NEs Downregulate AURKA and the PI3K/AKT/HIF-1α Pathway While Inhibiting Glycolysis-Related Enzyme Expression in OSCC

**Figure 5:**
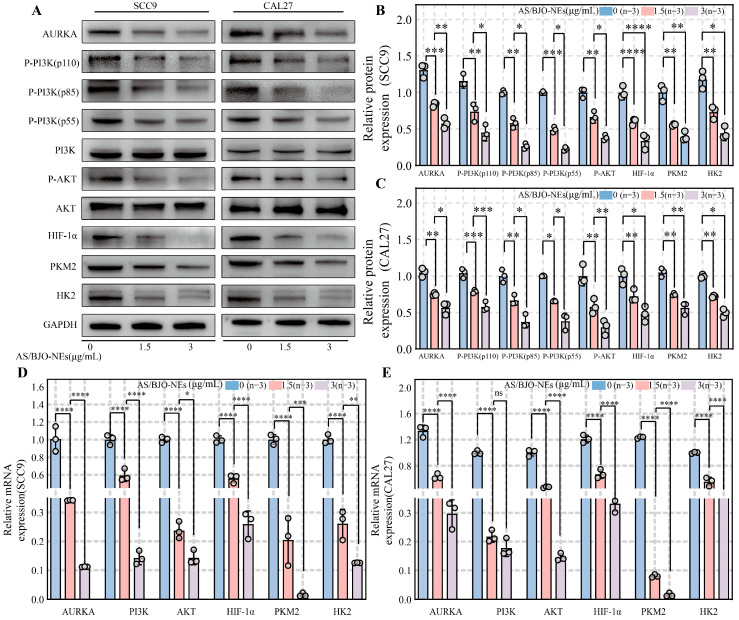
Western blot and RT-qPCR quantified protein levels and gene expression changes in target molecules following AS/BJO-NEs treatment: (**A**) Blot graph of Western blot results for each target protein against the internal reference protein. (**B**) Quantitative values of different protein expression of SCC9 cell line in Western blot under three conditions. (**C**) Quantitative values of different protein expression of CAL27 cell line in Western blot under three conditions. The 3 groups of conditions were AS/BJO-NEs (0, 1.5, 3 μg/mL) for 24 h. (**D**,**E**) RT-qPCR to detect the mRNA expression levels of relevant molecules, in two cell lines, SCC9 and CAL27, under different conditions (AS/BJO-NEs (0, 1.5, 3 μg/mL) for 24 h). “ns” is not significant, * *p* < 0.05, ** *p* < 0.01, *** *p* < 0.001, **** *p* < 0.0001. The circles on the bar chart represent the sample size or the number of repetitions, while the bars represent the mean ± SD. *n* = 3 indicates three repetitions for each group.

To further investigate the molecular role of AS/BJO-NEs, we conducted Western blotting and RT-qPCR analyses. Western blot analysis revealed that protein expression of AURKA, PI3K/AKT/HIF-1α pathway components, and glycolysis-related enzymes (HK2, PKM2) was significantly inhibited in both OSCC cell lines treated with low- or high-concentration AS/BJO-NEs, compared to untreated controls (Figure 5A–C). RT-qPCR showed that the mRNA expression of AURKA, PI3K, AKT, HIF-1α, HK2, and PKM2 was also inhibited (Figure 5D,E). These results demonstrate that AS/BJO-NEs downregulate AURKA expression and suppress the PI3K/AKT/HIF-1α pathway activity in OSCC, significantly inhibiting glycolysis in OSCC.

### 2.10. AS/BJO-NEs Suppress OSCC Proliferation, Invasion, Metastasis, and Glycolytic Activity by Targeting the AURKA/PI3K/AKT/HIF-1α Signaling Pathway

**Figure 6:**
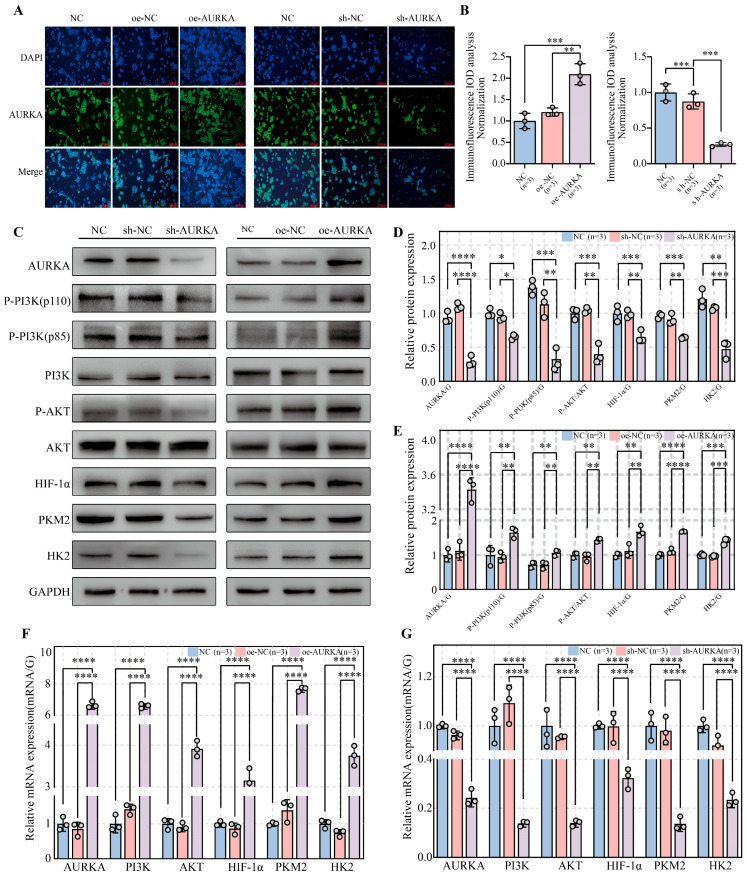
The impact on the pathway after establishing AURKA knockdown/overexpression cell lines: (**A**,**B**) Immunofluorescence assay to detect the changes in AURKA levels after overexpression (**A**) and knockdown (**B**) of AURKA. (**C**–**E**) Western blotting detected protein expression following AURKA knockdown (**C**, left) and overexpression (**C**, right). (**D**,**E**) demonstrate that AURKA processing markedly influences both pathway activation and glycolytic enzyme protein expression. Moreover, the plasmid does not have any impact on the subsequent experiments. (**F**,**G**) The RT-qPCR analysis measured mRNA expression levels following both AURKA overexpression (**F**) and knockdown (**G**). * *p* < 0.05, ** *p* < 0.01, *** *p* < 0.001, **** *p* < 0.0001. The circles on the bar chart represent the sample size or the number of repetitions, while the bars represent the mean ± SD. *n* = 3 indicates three repetitions for each group.

Immunofluorescence analysis (Figure 6A,B) revealed cytoplasmic localization of AURKA in OSCC cells, with overexpression significantly increasing and knockdown markedly decreasing its protein levels. On the other hand, Western blot (Figure 6C–E) and RT-qPCR analyses (Figure 6F,G) showed that AURKA overexpression activated the PI3K/AKT/HIF-1α signaling pathway and increased key glycolytic enzyme activity at both the protein and mRNA levels, whereas knockdown conversely suppressed these effects. These findings suggest that AS/BJO-NEs modulate this pathway and the OSCC glycolytic program primarily by downregulating AURKA, rather than by directly targeting its individual downstream components.

To further investigate the effects of AURKA and AS/BJO-NEs on the malignant biological behavior of OSCC, we established a control group (untreated cells) and several experimental groups (AURKA overexpression, knockdown, and their combinations with AS/BJO-NEs). The following assays were performed, including clone formation assay, transwell invasion assay, scratch assay, and glycolysis assay.

**Figure 7:**
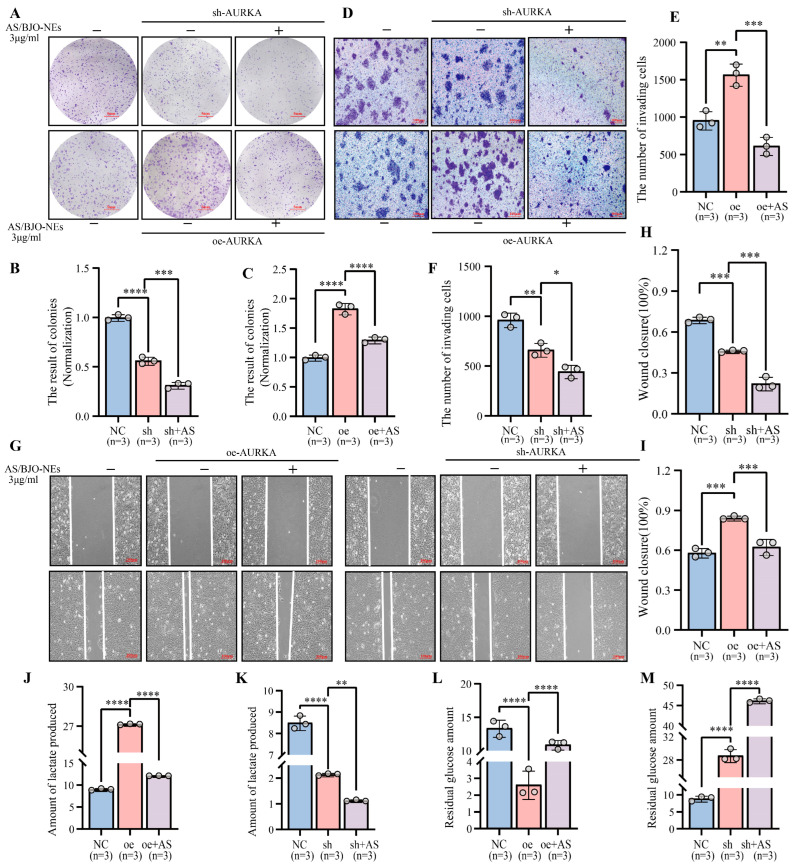
Impact of AURKA knockdown and overexpression on proliferation, migration, invasion, and glycolytic metabolism in OSCC cells: (**A**–**C**) Clone formation assays assessed how AURKA knockdown (**A**, upper panels; **B**) and AURKA overexpression (**A**, upper panels; **C**) affected OSCC cell proliferation, along with the modulatory role of AS/BJO-NEs in this process. (**D**–**F**) Transwell invasion assay investigated the effects of AURKA knockdown (in the lower part of **D**,**E**) and AURKA overexpression (in the upper part of **D**; **F**) on the invasion ability of OSCC cells, as well as the influence of AS/BJO-NEs in this process. (**G**–**I**) The scratch assay assessed how AURKA overexpression (in the left part of **G**; **H**) and AURKA knockdown (in the right part of **G**; **I**) affected OSCC cell migration and the influence of AS/BJO-NEs in it. (**J**,**K**) After AURKA overexpression, the lactate production of OSCC cells increased, while AS/BJO-NEs inhibited this promoting effect (**J**); after AURKA knockdown, the lactate production of OSCC cells decreased, while AS/BJO-NEs strengthened this inhibitory effect (**K**). (**L**,**M**) After overexpression of AURKA, the glucose uptake ability of OSCC cells was enhanced, while AS/BJO-NEs inhibited this promoting effect (**L**). After knockdown of AURKA, the glucose uptake ability of OSCC cells decreased, and AS/BJO-NEs strengthened this inhibitory effect (**M**). * *p* < 0.05, ** *p* < 0.01, *** *p* < 0.001, **** *p* < 0.0001. The circles on the bar chart represent the sample size or the number of repetitions, while the bars represent the mean ± SD. *n* = 3 indicates three repetitions for each group. “sh” or “oe” denotes the sh-AURKA or oe-AURKA group. “sh+AS” or “oe+AS” denotes the sh-AURKA+AS/BJO-NE or oe-AURKA+AS/BJO-NE group.

**Figure 8:**
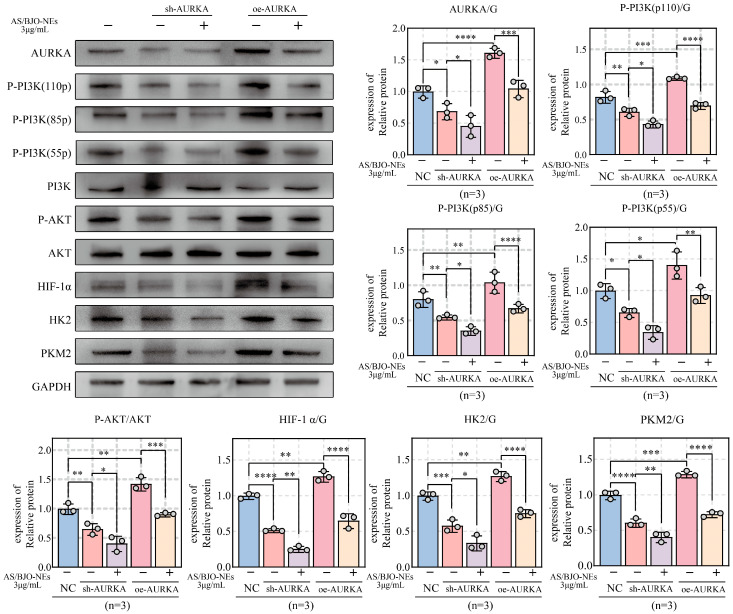
Western blot analysis assessed alterations in PI3K/AKT/HIF1α pathway protein expression and key glycolytic enzymes (PKM2, HK2) following AURKA knockdown or overexpression, as well as combined AS/BJO-NEs treatment in OSCC cells. * *p* < 0.05, ** *p* < 0.01, *** *p* < 0.001, **** *p* < 0.0001. The circles on the bar chart represent the sample size or the number of repetitions, while the bars represent the mean ± SD. *n* = 3 indicates three repetitions for each group.

Knockdown of AURKA markedly suppressed proliferation, invasion, migration, and glycolysis in OSCC cells, while AS/BJO-NEs further amplified these inhibitory effects (Figure 7A–I,K,M). However, in the AURKA overexpression group, OSCC cells exhibited a more aggressive malignant phenotype, though AS/BJO-NEs partially attenuated this phenotype enhancement (Figure 7A–I,J,L). In addition, the Western blot results for the above groups revealed that high AURKA expression enhanced the activation of the PI3K/AKT/HIF-1α pathway, consequently upregulating the glycolytic enzymes HK2 and PKM2. This effect was partially inhibited by the combination of AS/BJO-NEs. AURKA knockdown reduced expression of both pathway-related proteins and glycolytic enzymes, an effect further potentiated by the AS/BJO-NEs combination (Figure 8).

Thus, by combining bioinformatics results with in vitro experiments, we demonstrated that AS/BJO-NEs suppress OSCC proliferation, invasion, metastasis, and glycolytic metabolism via the AURKA-mediated PI3K/AKT/HIF-1α signaling pathway.

### 2.11. In Vivo Experimental Validation of AS/BJO-NEs Downregulates AURKA and Inhibits OSCC Proliferation and Glycolysis

**Figure 9:**
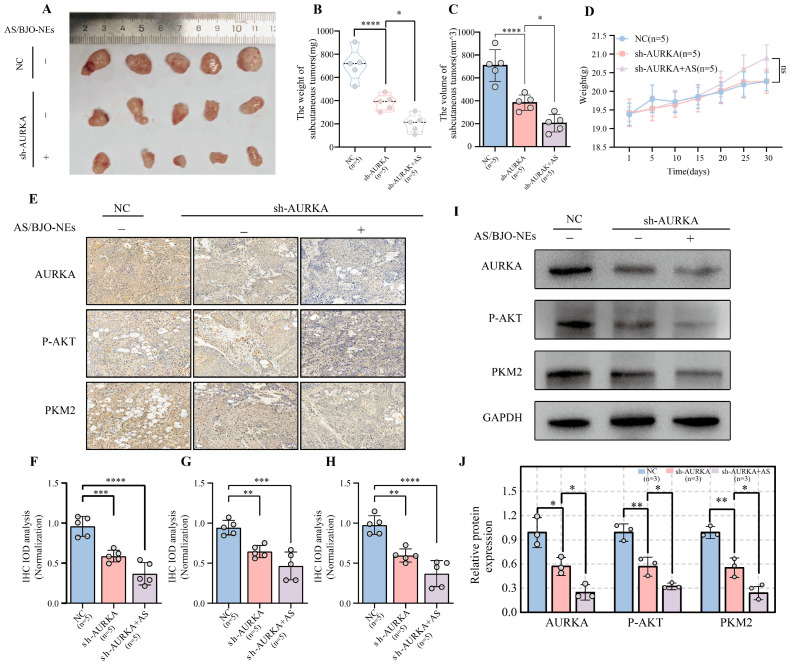
In vivo experiments were conducted to establish subcutaneous transplanted tumors in immunodeficient mice: (**A**) Images of subcutaneous tumors in each group. (**B**) The subcutaneous tumor weights differed significantly across the experimental groups. (**C**) The subcutaneous tumor volumes differed significantly across the experimental groups. (**D**) Quantitative graph of weight changes in each group of nude mice. (**E**) IHC staining. (**F**–**H**) The quantitative analysis diagrams of IOD for IHC. (**I**,**J**) After extracting proteins from tumors of different groups, Western blot detected the expression levels of AURKA, P-AKT, and PKM2 (**I**). (**J**) shows significant differences among different treatment groups. “ns” is not significant, * *p* < 0.05, ** *p* < 0.01, *** *p* < 0.001, **** *p* < 0.0001. The circles on the bar chart represent the sample size or the number of repetitions, while the bars represent the mean ± SD. *n* = 3 or *n* = 5 indicates three repetitions or five samples for each group.

OSCC subcutaneous graft tumor nude mouse models were established using normal OSCC cell lines as well as knockdown cell lines. Compared with the NC group (0.9% saline solution), subcutaneous tumor growth was significantly suppressed in both the sh-AURKA group and the combination treatment group (sh-AURKA+ AS/BJO-NE group), with a more pronounced inhibitory effect observed in the combination group (Figure 9A–C). Specifically, compared to the NC group, the tumor volume reduction rates were 60% in the sh-AURKA group and 79% in the sh-AURKA+ AS/BJO-NE group. There was no significant difference in the weight changes in mice among different groups (Figure 9D). Furthermore, IHC staining revealed reduced expression of AURKA, P-AKT, and PKM2 in the AS/BJO-NE group, indicating that AS/BJO-NEs can downregulate AURKA and the mediated pathways with glycolytic metabolic processes (Figure 9E–H). We also confirmed this through Western blot experiment in which proteins were extracted from the transplanted tumor tissues (Figure 9I,J).

In conclusion, both in vivo and in vitro experiments confirmed that AS/BJO-NEs suppress OSCC proliferation, invasion, metastasis, subcutaneous tumorigenesis, and glycolytic metabolism through targeting the AURKA/PI3K/AKT/HIF-1α pathway.

## 3. Discussion

OSCC is the most prevalent type of squamous cell carcinoma affecting the head and neck region [27]. Cases of OSCC account for an excess of 90% of the approximately 355,000 new cases of oral cancer diagnosed each year [28]. Notwithstanding the continuous advancements in surgical techniques, adjuvant treatments, and research on the pathogenesis of OSCC, patients continue to experience high recurrence rates and poor prognosis [3]. Compared with paraneoplastic normal epithelial cells, OSCC malignant cells show a significant Warburg effect feature, which is an important reason for promoting cell invasion and metastasis and affecting patient prognosis [5]. TCM has multi-target and multi-pathway characteristics, which enable it to exert anti-tumor effects by regulating aerobic glycolysis and energy metabolism [9,29]. Therefore, searching for efficient anti-tumor drugs in TCM is a novel approach to the treatment of OSCC. We prepared AS-IV in combination with BJO to form AS/BJO-NEs. This new type of nanoemulsion combines two Chinese medicine ingredients with nanotechnology. It is designed to inherit the synergistic therapeutic advantages of TCM, while enhancing drug efficacy through nanotechnology.

The experiments conducted in this study demonstrated that the growth of OSCC cells was significantly inhibited by AS/BJO-NEs. Furthermore, the results showed a dose-dependent diminution in OSCC cell migration and invasion, as well as a decline in glucose uptake capacity and lactate production. However, the mechanism by which AS/BJO-NEs target the glycolysis of OSCC to inhibit their development is unclear. Therefore, we combined multi-omics methods, such as network pharmacology, machine learning, and single-cell genomics, with further in vitro and in vivo experiments to explore the mechanism by which AS/BJO-NEs regulate OSCC by targeting glycolytic metabolism.

Firstly, we constructed a glycolysis-related machine learning risk prognostic model for OSCC. From the above results, the glycolysis-hub genes (GHGs) targeted by AS/BJO-NEs for OSCC were obtained. In addition, by WGCNA, we further obtained glycolysis-related genes (GRGs) of AS/BJO-NEs targeting OSCC. In this process, we considered not only whether AS/BJO-NEs target machine learning model genes for the poor prognostic risk model of glycolysis, but also whether they target genes that are significantly associated with high-risk models, which considered a wider range. The acquisition of GHGs and GRGs enabled us to provide more possible options for the target study of AS/BJO-NEs in regulating OSCC glycolysis and to enrich the target research methods of conventional machine learning combined with network pharmacology. Finally, we determined that AURKA is a key target of AS/BJO-NEs to regulate glycolytic metabolism in OSCC. Molecular docking analysis demonstrated the binding stability between AS/BJO-NEs and AURKA, further corroborating the selection of the target genes.

AURKA is a serine/threonine kinase, which has been implicated in the initiation of various tumor types, and its aberrant activation is an important driver of the malignant phenotype of tumors [30]. Pan-carcinoma differential expression analysis of AURKA also demonstrates that it is highly expressed across a wide spectrum of tumor types. Our transcriptome-based analysis of OSCC indicates that AURKA exerts a significant impact on tumor development and patient outcome. Through in vitro experiments, we demonstrated that AURKA expression modulates the malignant phenotype of OSCC. Specifically, AURKA-overexpressing cells exhibited significantly enhanced proliferation, invasion, and migration. These findings align with the poorer prognosis observed in patients with high AURKA expression identified in our transcriptome analysis.

The present study also revealed that high AURKA expression was positively correlated with glycolytic metabolism and PI3K/AKT/mTOR activation in OSCC, which was further confirmed by transcriptome GSVA and single-cell transcriptomic analyses of pathways and metabolism. These findings strongly suggest that targeting AURKA to regulate glycolytic metabolism is a key mechanism through which AS/BJO-NEs exert their anti-OSCC effects. In addition, previous studies demonstrate that the AURKA inhibitor alisertib could inhibit the growth of HCC and neuroblastoma in vivo via the AURKA-AKT axis [31,32], and PI3K/AKT activation directly influences HIF-1α-regulated glycolytic pathways and accelerates malignant progression [33,34]. Therefore, AS/BJO-NEs likely inhibits glycolysis through the AURKA-mediated PI3K/AKT/HIF-1α axis, ultimately suppressing malignant progression of OSCC.

In this study, knockdown of AURKA downregulated protein expression and mRNA levels of PI3K/AKT/HIF-1α pathway components and glycolysis-related enzymes, while AURKA overexpression exerted the opposite effects. AS/BJO-NEs treatment inhibited the promotional effects of AURKA overexpression on both the pathway and glycolysis, while enhancing the inhibitory effects of AURKA knockdown. In vivo experiments, tumors in nude mice exhibited significantly slower growth in the sh-AURKA group than in the untreated controls. Furthermore, the addition of AS/BJO-NEs enhanced this tumor suppression effect. Phospho-AKT (p-AKT) protein expression can serve as a marker for PI3K/AKT pathway activation [35]. IHC analysis of tumor tissues revealed that not only AURKA but also p-AKT was suppressed following AS/BJO-NEs treatment. Concurrently, expression of the glycolysis-related enzyme was also markedly inhibited across the different treatment groups. Collectively, both in vitro and in vivo experiments demonstrated that AS/BJO-NEs downregulated the glycolysis level through the AURKA-mediated PI3K/AKT/HIF-1α pathway, thereby inhibiting the proliferation, invasion, and metastasis of OSCC.

The results of the current study also indicate a correlation between AURKA expression and the immune microenvironment in OSCC. The elevated AURKA expression correlated with higher TIDE scores, which are associated with reduced sensitivity to ICB therapy [36]. Immune checkpoint correlation analysis showed that high AURKA expression promoted the expression levels of CD276, CD44, HHLA2, and LAG3. These molecules are associated with immune evasion and play a significant role in cell proliferation, invasion, and migration in malignant tumors [37,38,39,40]. In the immune infiltration analysis, the high expression of AURKA inhibits IDC, Mast cell, Tfh cell, Plasmacytoid dendritic cell (pDC), and promotes Activated CD4 T cell, Th2 cell. The balance of immune cells within the TME is critical [41]. Within the tumor immune microenvironment (TME), inhibition of IDCs reduces the number of dendritic cells (DCs) maturing and impedes their migration to lymph nodes for antigen presentation to T cells [42,43]. Th2 cells can activate M2 macrophages and secrete inflammatory factors such as IL-4 and IL-5, which promote tumor progression [41]. Furthermore, in many solid tumor types, an increased frequency or enhanced function of Tfh cells is often associated with improved anti-tumor immunity [44,45]. Moreover, OSCC patients with low mast cell density exhibited poorer overall survival, indicating that low mast cell density is also associated with adverse tumor outcomes [46,47].

Therefore, AS/BJO-NEs also improve the tumor microenvironment to some extent, and demonstrate anti-inflammatory potential through the inhibition of inflammatory factor release from immune cells. This characteristic was also demonstrated in the enrichment analysis of the network pharmacology. KEGG enrichment analysis revealed that the anti-OSCC targets of AS/BJO-NEs were significantly enriched in multiple signaling pathways, including TNF, JAK-STAT, FOXO, and MAPK pathways, among others. These pathways are closely related to inflammatory responses and oxidative stress. For instance, the JAK-STAT pathway is related to the inflammatory response mediated by cytokines like IL-2, IL-15, and IFN, and its excessive activation can promote tumor development [48]. Downregulating the FOXO pathway can elevate intracellular ROS levels and accelerates tumor progression [49]. This multi-faceted and multi-pathway coordinated anti-OSCC effect may be attributed to its principal constituents, AS-IV and BJO, both of which exhibit anti-inflammatory and antioxidant activities. Specifically, AS-IV exerts anti-inflammatory effects by targeting C5a and C5aR1, and also mitigates oxidative stress via the FOXO pathway [50,51]. BJO, on the other hand, which can not only attenuate oxidative stress by upregulating superoxide dismutase and reducing Malondialdehyde levels, but can also synergistically exert antioxidant, anti-inflammatory, and anti-tumor effects by downregulating pro-inflammatory mediators such as IL-8 and VEGF [52,53].

These findings clearly indicate that the AS/BJO-NEs not only inhibit glycolytic metabolism, proliferation, invasion, and metastasis of OSCC by downregulating the AURKA-mediated PI3K/AKT/HIF-1α pathway, but may also exert anti-inflammatory and antioxidant effects through multiple targets and pathways to suppress tumor progression. This demonstrates more possibilities for AS/BJO-NEs in their research on combating OSCC through multiple targets and pathways, while also broadening our thinking perspectives and conceptual framework for enhancing their anti-tumor efficacy in future research.

## 4. Materials and Methods

### 4.1. Network Pharmacological Analysis of AS/BJO-NEs Against OSCC

The active ingredients with oral bioavailability (OB) ≥ 30 and drug-likeness (DL) ≥ 0.18 in YA DAN ZI (BJO) were obtained from the TCMSP database (https://tcmsp-e.com/tcmsp.php, accessed on 9 September 2024). Chemical information related to the active ingredients was obtained through the Pubchem database (https://pubchem.ncbi.nlm.nih.gov/, accessed on 9 September 2024), and the SwissTargetPrediction platform (http://www.swisstargetprediction.ch/, accessed on 9 September 2024) was used to obtain predicted targets (score > 0). OSCC-related target genes were obtained from the OMIM database (http://www.omim.org/, accessed on 9 September 2024), the GeneCards database (https://www.genecards.org/, accessed on 9 September 2024), the DisGeNET database (http://www.disgenet.org/), the TDD database (https://db.idrblab.net/ttd/, accessed on 9 September 2024) and the PharmGKB (https://www.pharmgkb.org/, accessed on 9 September 2024).

The drug targets and OSCC target genes were normalized by UniProt database (https://www.uniprot.org/, accessed on 9 September 2024), the unmapped targets were removed, and the intersecting targets were obtained by “ggvenn” R package (version 0.1.10) as the candidate targets of AS/BJO-NEs against OSCC. Finally, a visual network was constructed using the STRING database (https://cn.string-db.org/, accessed on 9 September 2024) and Cytoscape software (version 3.10.2).

### 4.2. Obtain Transcriptome and Single-Cell Transcriptome Data

OSCC transcriptome datasets, GSE30784, GSE41613, GSE65858, and GSE85446, along with their related clinical information were obtained from the GEO database (https://www.ncbi.nlm.nih.gov/geo, accessed on 18 October 2024) (Supplementary Table S8). Concurrently, RNA-Seq data and clinical data from TCGA-HNSCC patients were procured from the TCGA database (https://portal.gdc.cancer.gov/, accessed on 18 October 2024). Samples originating from the oral cavity were retained on the basis of the clinical data (Supplementary Table S8). TCGA-Pan-Cancer transcriptome data were obtained from the UCSC Xena database (https://xenabrowser.net/datapages/, accessed on 18 October 2024) (Supplementary Table S8). Single-cell analysis data were obtained from GSE188737 (including 7 HNSCC primary foci with 7 HNSCC lymphatic metastasis samples).

### 4.3. Construction of a Machine Learning Prognostic Risk Model Related to OSCC Glycolysis

The glycolysis-related gene set was obtained from the GSEA database (https://www.gsea-msigdb.org/gsea/msigdb/index.jsp, accessed on 18 October 2024) (Supplementary Table S2), and the genes related to survival prognosis were then screened from the gene set as feature genes for machine learning model analysis.

RNA-Seq data and clinical survival data from TCGA-OSCC as well as GSE41613 were collated, with the former serving as the training set (OSCC samples) and the latter as the validation set for the machine learning model. Combined with the aforementioned feature genes, the TCGA training set and the GEO validation set were used to construct an OSCC glycolysis-related machine learning prognostic risk model employing a combination of algorithms in R language [54]. The result of the Kaplan–Meier analysis of the optimal risk model is visualized using the “survival” package (version 3.7.0).

### 4.4. Unsupervised Cluster Analysis Based on Machine Learning Model Genes

Cluster analysis of OSCC transcriptome samples was performed using expression data of resultant genes from machine learning prognostic analysis models by the “ConsensusClusterPlus” R package (version 1.68.0), obtaining the optimal results and outputting the results.

### 4.5. Weighted Gene Co-Expression Network Analysis (WGCNA)

WGCNA is able to analyze associations between gene modules and biological traits to identify hub genes with strong phenotypic relevance. The transcriptome dataset was analyzed using the “WGCNA” R package (version 1.72.5) to identify differentially expressed genes. Modules were defined with a minimum size of 50 and a cutting height of 0.25. The correlation matrix between the gene consensus modules and the corresponding phenotypes was established to obtain the core genes that were strongly associated with the relevant phenotypes.

### 4.6. Function Enrichment Analysis

KEGG and GO enrichment analysis were performed and visualized using “clusterProfiler” R package (version 4.12.0) and “ggplot2” R package (version 3.5.1). GSEA analysis gene sets (h.all.v2024.1.Hs.symbols.gmt) (Supplementary Table S2) were obtained through the GSEA database using the R language “GSEA” software package (version 4.12.0) for GSEA analysis.

### 4.7. Immune Infiltration and TIDE Analysis

Immune cell abundance was quantified from transcriptomic data using ssGSEA and GSVA algorithms implemented in R, while expression levels of immune checkpoint genes were concurrently extracted. The transcriptome samples were stratified based on core gene expression to examine variations in immune infiltration and immune checkpoint expression across distinct gene expression levels in OSCC.

TIDE can assess immune escape in the tumor immune microenvironment based on transcriptomic data. The higher the TIDE score, the more likely immune escape is to occur, and the lower the likelihood of immunotherapy benefit [36]. The OSCC transcriptome dataset was scored using the R language and the TIDE platform (http://tide.dfci.harvard.edu/, accessed on 10 January 2025) to evaluate the differences in immune response of OSCC samples under different treatments.

### 4.8. Drug Sensitivity Analysis

The “oncoPredict” package (version 1.2.0) and the GSCA platform (https://guolab.wchscu.cn/GSCA/#/, accessed on 10 January 2025) were used to analyze the drug sensitivity of OSCC transcriptomic data, evaluate the tolerance of OSCC samples to anticancer drugs under different conditions by different group treatments, and analyze the relationship between gene expression and drug sensitivity.

### 4.9. Molecular Docking

Based on the network analysis of the AS/BJO-NEs–target-OSCC network diagram in Cytoscape, specific ligands were selected for subsequent operations. These included AS-IV and the active ingredients from BJO that target AURKA, along with the other top five active ingredients identified in the network analysis. All compounds were downloaded via the TCMSP database (Supplementary Table S9).

The atomic coordinates of AURKA were obtained from the Protein Data Bank (PDB, https://www.rcsb.org/, accessed on 10 January 2025) (Filter criteria: Homo sapiens was selected for Scientific Name of Source Organism; X-RAY DIFFRACTION was selected for Experimental Method) and processed in AutoDock4.

Finally, the binding interactions of the ligand–receptor complex were visualized using PyMOL, while binding energy data were analyzed and plotted with the “pheatmap” package (version 1.0.12) in R 4.4.1.

### 4.10. The Composition and Preparation of AS/BJO-NEs

The materials required for preparation include 0.6 mL BJO, 0.06 g AS-IV, 10 g soybean oil, 0.79 g soybean phospholipids, and 1.4 g glycerol. After preparation, BJO and AS-IV are dissolved in heated soybean oil to form the oil phase. Glycerol and soybean phospholipids are stirred and dissolved in ultrapure water to form the water phase. The two phases are mixed using a high shear disperser at a temperature of 70 °C, then slowly added to the oil phase at a speed of 13,000 rpm (taking 5 min), followed by high-speed shearing at 19,000 rpm for 5 min to obtain the pre-milk. The pre-milk is finally homogenized 15 times at 40–50 °C and 538 bar pressure using a high-pressure homogenizer (Antuosi Nanotechnology Co., Ltd, Suzhou, China) to obtain the AS/BJO-NEs nanoemulsion. The particle size, PDI, and potential of AS/BJO-NEs were measured using the Malvern ZS 90 laser particle size analyzer (Malvern Instruments Limited, Malvern, United Kingdom) to evaluate the characterization of AS/BJO-NEs.

### 4.11. UPLC/MS Spectrum

Chromatographic conditions:

Separation was performed on a Thermo Scientific C18 column (4.6 mm × 250 mm, 5 μm) maintained at 30 °C. The mobile phase consisted of water and acetonitrile (60:40, *v*/*v*) delivered at a flow rate of 1.0 mL/min. The injection volume was 30 μL, and the total run time was 20 min. Detection was carried out at 203 nm using an evaporative light scattering detector (ELSD, Shimadzu Corporation, Kyoto, Japan) with the drift tube temperature set at 40 °C.

Mass spectrometry conditions:

Analysis was conducted using electrospray ionization (ESI) in positive ion mode with multiple reaction monitoring (MRM). The ion spray voltage was 5500 V and the ion source temperature was 550 °C. Nitrogen was used as the nebulizing gas (55 psi), auxiliary gas (55 psi), and curtain gas (20 psi). Reaction pairs: Astragaloside IV m/z 823.5→643.5. The collision energy (CE) for Astragaloside IV is 65 V, and the dissociation voltages (DP) is 100 V. Please refer to “Supplementary Table S10” for details of all purchases and sources in the subsequent descriptions.

### 4.12. Cell Culture and Manipulation

OSCC cell lines (CAL27, SCC9) and Human oral mucosal keratinocyte (HOK) cells were selected and routinely cultured in DMEM medium containing 10% fetal bovine serum (FBS), 100 U/mL penicillin, and 100 μg/mL streptomycin at 37 °C in a 5% CO_2_ incubator. Cells in the logarithmic growth phase were used for subsequent experiments.

### 4.13. Establish AURKA Knockdown/Overexpression Cell Lines

Construct the lentiviral vector containing AURKA-cDNA with an empty multiple cloning site (MCS) control and the lentiviral vector containing AURKA-shRNA with an empty control, in order to achieve AURKA overexpression (oe-AURKA) and knockdown (sh-AURKA). Then, clone the AURKA-cDNA or the specific shRNA sequence into the lentiviral vector, and co-transfect it with the helper plasmids psPAX2 and pMD2.G into HEK 293T cells. The viral supernatant was collected and concentrated by centrifugation, after which the viral titer was detected by gradient dilution. CAL27 cells were infected at different concentrations, and the optimal conditions were determined based on fluorescence intensity. Determine the target cell’s puromycin lethal concentration, obtain stably expressing oe-AURKA and sh-AURKA cell lines, and verify the success of the constructs using RT-qPCR and Western blot.

### 4.14. Cell Counting Kit-8 Assay and Colony Formation

CAL27, SCC9 cells, and HOK cells in logarithmic growth phase were inoculated into 96-well plates at a density of 5000 cells per well, with five replicate wells and blank wells, and incubated in a cell culture incubator for 24 h. Different concentrations of AS/BJO-NEs (2 μg/mL, 4 μg/mL, 6 μg/mL, 8 μg/mL, 10 μg/mL) were added and further incubated in the incubator for 24 h. After successful modeling, serum-free medium containing 10 μL CCK8 solution was added to each well, and the optical density was detected by measuring the absorbance value at 450 nm using a multifunctional enzyme marker to derive the proliferative capacity of the cells. The inhibition rate of each group of cells was calculated from the absorbance value. The percentage of cell viability was calculated using the following formula:

$$Cell\;viability\left(\%\right)=\frac{OD\left(treatment\right)-OD\left(blank\right)}{OD\left(control\right)-OD\left(blank\right)}\times \%$$

On the other hand, for colony formation, 5000 cells were seeded in a 6-well plate and the medium was changed every 5 to 7 days. Colonies were stained with 0.1% crystal violet after 14–28 days. The experiment was repeated 3 times. Calculate the statistical significance using one-way ANOVA.

### 4.15. Cell Scratch Test

Draw even horizontal lines on the back of the 6-well plate at 0.5–1 cm. Seed each well with 2 mL of OSCC cell suspension (5 × 10^5^ cells/mL) and place in a constant temperature and humidity incubator (Panasonic Healthcare Holdings Co., Ltd. Osaka, Japan) at 37 °C, 5% CO_2_ for 24 h. The control group was given 2 mL of medium, and the experimental group was given 1.5 µg/mL and 3 µg/mL of medium containing AS/BJO-NEs, respectively. The migration of the cells was observed under the microscope (Leica Microsystems, Wetzlar, Germany) and photographed and recorded at 0 and 48 h, respectively. The migration rate was calculated using the following formula:

$$Migration\;rate\left(\%\right)=\frac{scratch\;area\left(0\;h\right)-scratch\;area\left(48\;h\right)}{scratch\;area\left(0\;h\right)}\times \%$$

The experiment was repeated 3 times. Calculating the statistical significance using one-way ANOVA.

### 4.16. Transwell Invasion Assay

Dilute matrix gel with serum-free at a 1:8 ratio. Spread it on the membrane of the transwell chamber and incubate at 37 °C to solidify. Then hydrate for 2 h. Add 200 μL cell suspension (5 × 10^5^ cells/mL) to the upper chamber and 750 μL DMEM medium containing 10% FBS to the lower chamber. Continue the incubation in the cell incubator for 24 h. After incubation, remove the transwell as well as the upper and lower media. Wash the cells three times with PBS. Fix with 4% paraformaldehyde and stain with 0.1% crystal violet. And place under an inverted fluorescence microscope (Leica Microsystems, Wetzlar, Germany) for observation. Take three fields of view randomly for photographs, and count the number of cells passing through the cell chambers. The experiment was repeated 3 times. Calculate the statistical significance using one-way ANOVA.

### 4.17. Western Blot

Lysates were prepared using RIPA, PMSF, and phosphorylated protease inhibitors, and each group of cells was lysed, then the cells were collected and centrifuged. Collect the supernatant. Determine the protein concentration of the sample using the BCA kit (Solarbio, Beijing, China). Samples were spiked according to each grouping of proteins and separated by 10% SDS-PAGE electrophoresis, followed by constant-pressure membrane transfer to PVDF membranes. The proteins were incubated with the specific primary antibody and the appropriate secondary antibody. The blots were then detected using the UVP ChemStudio-515 imaging system (Analytik Jena GmbH, Upland, CA, USA). The experiment was repeated 3 times. After normalizing the data results, a one-way analysis of variance was used to calculate the statistical significance.

### 4.18. Reverse Transcription Quantitative Polymerase Chain Reaction (RT-qPCR)

Process in groups, extract total RNA using standard reagents, check the purity and concentration, and then remove genomic DNA and synthesize cDNA. The amplification process was conducted within a real-time fluorescence quantitative PCR system with appropriate cycling conditions set. GAPDH served as the internal reference gene, corrected for amplification efficiency (90–105%), and the experiment was set up with three technical replicates and biological replicates to test and analyze the differences between the groups. (Primers and conditions are shown in “Supplementary Table S11”). The experiment was repeated 3 times. After normalizing the data results, a one-way analysis of variance was used to calculate the statistical significance.

### 4.19. Immunofluorescence Assay (IFA)

Conduct experiments on cells that grow logarithmically. First, immerse the slides in 75% alcohol for soaking. Place them in a sterile Petri dish with sterile forceps. Then, wash away the excess alcohol with PBS and place the cells in a culture box for further overnight culture, at 4% paraformaldehyde fixation. Permeabilization of 0.5% Triton X-100 for 20 min was performed, and serum was blocked at room temperature for 30 min. Primary antibody was incubated at 4 °C overnight. DAPI staining was performed after secondary antibody incubation, and images were collected and observed under fluorescence microscope. The experiment was repeated 3 times. Calculate the statistical significance using one-way ANOVA.

### 4.20. Glycolytic Metabolite Assays

Cells were inoculated for growth in 6-well plates and later incubated in groups for 24 h under different conditions. Then, use the Glucose Assay Kit (Nanjing Jian Cheng Bioengineering Institute, Nanjing, China) and Lactate Assay Kit (Nanjing Jian Cheng Bioengineering Institute, Nanjing, China) according to the kit instructions, collect the cell culture medium and test for glucose and lactate. The experiment was repeated 3 times. Calculate the statistical significance using one-way ANOVA.

### 4.21. Animal Experiments

Four- to six-week-old BAIB /c-nu nude female mice of SPF grade were selected and randomly assigned to experimental groups. Following one week of routine rearing, the mice were anesthetized with ether and received a dorsal injection of either AURKA wild-type or AURKA-downregulated OSCC cells (2 × 10^6^ cells/mL) to establish a subcutaneous xenograft tumor model. When the tumor volume reached approximately 100 mm^3^, the mice received daily intraperitoneal injections of either different doses of AS/BJO-NEs (50 mg/kg or 100 mg/kg) or 0.9% normal saline. After seven weeks of treatment, all nude mice were executed, and tumor tissues were excised, weighed, photographed, and processed for follow-up experiment. Tumor volume was calculated using the following formula:

$$V=\frac{length\times {\mathrm{width}}^{2}}{2}$$

Each group should have at least 3 samples. The statistical significance of the relevant parameters is calculated using one-way ANOVA. The Medical Ethics Committee of Jiangxi University of Chinese Medicine approved our study.

### 4.22. Immunohistochemical (IHC) Staining

The transplanted tumors from different groups of nude mice in the animal experiments were obtained for immunohistochemical detection after the tumor tissue blocks were sectioned, dehydrated, and rehydrated, and heated. After being incubated at room temperature with 3% FBS for 1 h, different sections were incubated overnight at 4 °C with anti-AURKA, anti-P-AKT, and anti-PKM2 antibodies, washed, and then incubated with secondary antibodies. The sections were observed under a microscope (Leica Microsystems, Wetzlar, Germany) after staining.

### 4.23. Statistical Analysis

For survival analysis, ROC (Receiver Operating Characteristic) analysis was statistically analyzed by Logrank test using R 4.4.1 software, and gene expression difference analysis was analyzed by *t*-test. Univariate and multivariate regression analysis was performed through simple and multiple linear regression in R software. Experimental results were statistically analyzed and data were visualized using GraphPad Prism 8.0 software. All experimental data are means ± SD of at least three independent experiments, independent sample *t*-tests were used for quantitative comparisons between two groups, and one-way ANOVA was used for quantitative comparisons between multiple groups; p-values < 0.05 indicate statistical significance. “ns” is not significant, * *p* < 0.05, ** *p* < 0.01, *** *p* < 0.001, **** *p* < 0.0001.

## 5. Conclusions

In summary, in this study, by combining network pharmacology, machine learning algorithms, and multi-omics analysis, we screened the glycolytic hub genes and glycolysis-related genes targeted by AS/BJO-NEs, and revealed the potential molecular mechanism by which AS/BJO-NEs inhibit glycolysis and thus act as an anti-OSCC through the AURKA-mediated related pathway. We also conducted in vitro and in vivo experiments, which demonstrated that AS/BJO-NEs exert anti-OSCC effects by downregulating AURKA, decreasing PI3K/AKT/HIF-1α-mediated OSCC glycolytic metabolism, and inhibiting OSCC proliferation, invasion, and metastasis. These findings lay the foundation for the multi-target and multi-pathway anti-OSCC study of AS/BJO-NEs. AS/BJO-NEs are expected to be a potential adjuvant therapeutic drug for the treatment of oral cancer and provide a new option for the clinical treatment of oral cancer. Furthermore, this study suggests that targeting AURKA-mediated glycolytic metabolism may become a new strategy for anticancer.

## Data Availability

The original contributions presented in this study are included in the article/Supplementary Material. Further inquiries can be directed to the corresponding author.

## Supplementary Materials

The following supporting information can be downloaded at https://www.mdpi.com/article/10.3390/ph18121783/s1, Table S1: The relevant targets obtained through network pharmacology. Table S2: Enrichment analysis gene set and univariate Cox analysis of glycolysis gene set. Table S3: The results obtained by different machine learning algorithms. Table S4: DEGs among different subtypes of glycolysis (C1 and C2) in TCGA-OSCC and GSE41613. Table S5: Differentially expressed genes (DEGs) between the OSCC group and the control group in TCGA-OSCC dataset. Table S6: Correlation analysis of AURKA with other genes in the transcriptome dataset. Table S7: Inhibition rate of AS/BJO-NEs on OSCC under different components. Table S8: Transcriptome dataset sample information. Table S9: The ligand for molecular docking. Table S10: The purchasing methods and sources required for the experiment. Table S11: PCR primers and reaction system. Figure S1: Pharmacological network analysis of AS/BJO-NEs against OSCC. Figure S2: Supplementary analysis of machine learning and cluster analysis. Figure S3: External validation of model genes. Figure S4: WGCNA of machine learning model validation set GSE41613. Figure S5: Survival analysis and ROC analysis of the GHGs and GRGs targeted by AS/BJO-NEs. Figure S6: Correlation analysis and ROC analysis of the GHGs and GRGs targeted by AS/BJO-NEs with glycolytic metabolic levels. Figure S7: Optimal truncation and supplementary bioinformatics analysis of AURKA. Figure S8: Supplementary validation of GSEA analysis. Figure S9: Specificity examination of UPLC/MS spectra. Figure S10: CCK-8 supplementation experiment. Figure S11: The effects of AS/BJO-NEs on subcutaneous transplanted tumors.

## Abbreviations

The following abbreviations are used in this manuscript:

OSCC	Oral squamous cell carcinoma
AS-IV	Astragaloside-IV
BJO	Brucea javanica oil
AS/BJO-NEs	Astragaloside–Brucea Javanica Oil nanoemulsion
TCM	Traditional Chinese Medicine
PPI	Protein–protein interaction
WGCNA	Weighted gene co-expression network analysis
GSEA	Gene Set Enrichment Analysis
GSVA	Gene Set Variation Analysis
GO	Gene Ontology
KEGG	Kyoto Encyclopedia of Genes and Genomes
HOK	Human oral mucosal keratinocytes
ROC	Receiver operating characteristic
GHGs	Glycolytic hub genes
GRGs	glycolysis-related genes
HK	Hexokinase
PKM2	Pyruvate Kinase M2
OB	Oral bioavailability
TME	Tumor immune microenvironment
DL	Drug-likeness
P-AKT	Phospho-AKT
Phospho-PI3K	P-PI3K
RT-qPCR	Reverse Transcription Quantitative Polymerase Chain Reaction
IFA	Immunofluorescence assay
IHC	Immunohistochemical
CCK8	Cell Counting Kit-8 assay
