# Supplementary material for: Astragaloside–Brucea Javanica Oil Nanoemulsion Regulates Glycolysis in Oral Squamous Cell Carcinoma Through AURKA-Mediated PI3K/AKT/HIF-1α Pathway
Source: Pharmaceuticals (Basel). 2025 Nov 24;18(12):1783. doi: 10.3390/ph18121783 (PMC12736130; doi:10.3390/ph18121783)
Supplement: Supplementary file 1 [file pharmaceuticals-18-01783-s001.zip › Supplementary Figures.pdf]

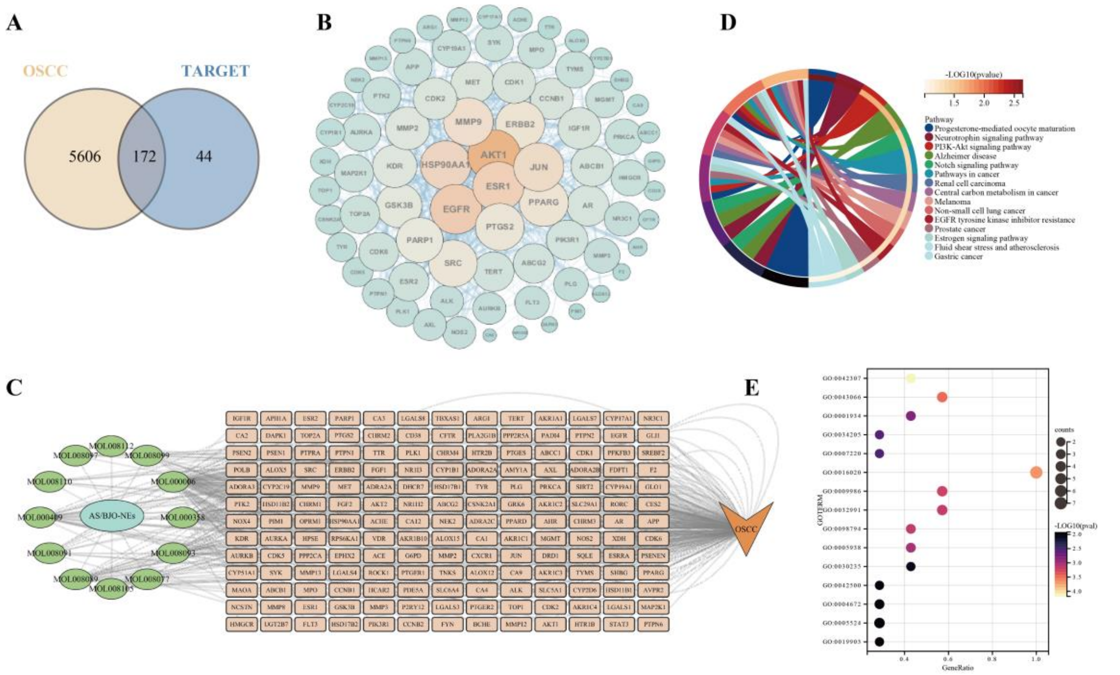

Figure S1: Pharmacological network analysis of AS/BJO-NEs against OSCC:(A)172 overlapping targets obtained by Venn analysis of the predicted targets of the two major components of AS/BJO-NEs and the OSCC targets;(B)visualisation of the PPI analysis of overlapping targets; (C)Visualisation of the drug-target-disease network diagram of AS/BJO-NEs - overlapping targets - OSCC;(D)Visualisation of the overlapping KEGG pathway enrichment analysis of the overlapping targets; (E)GO enrichment analysis of the overlapping targets.

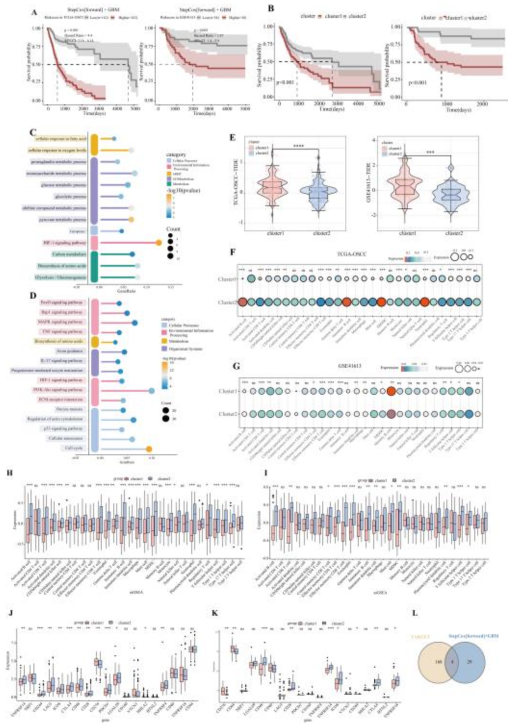

Figure S2:Supplementary analysis of machine learning and cluster analysis:(A)Survival analysis of training and validation set risk scores in the prognostic risk model of the StepCox[forward]+GBM

algorithm.(B)Survival analysis between the C1 and C2 subgroups of the glycolysis model gene risk in TCGA-OSCC and GSE41613.(C-D)Partial results of KEGG, GO analyses visualized for differential genes between C1 and C2 in the training and validation sets.(E)Figure show the difference in TIDE between the two subclasses of TCGA-OSCC and GSE41613.(F-G)F Graph shows immune cell differences in ssGSEA for the two subclasses TCGA-OSCC. G Graph shows immune cell differences in ssGSEA for the two subclasses GSE41613.(H)Visualisation box line plot of ssGSEA immune infiltration analysis between C1 and C2 of the TCGA-OSCC dataset; (I)Visualisation box line plot of ssGSEA immune infiltration analysis between C1 and C2 of the GSE41613 dataset; (J)Visualisation box line plot of immune checkpoint expression between C1 and C2 of the TCGA-OSCC dataset; (K)Visualisation box line plot of immune checkpoint expression between C1 and C2 of the GSE41613 dataset; (L)Venn analysis of AS/BJO-NEs anti-OSCC intersection targets with machine learning model genes.

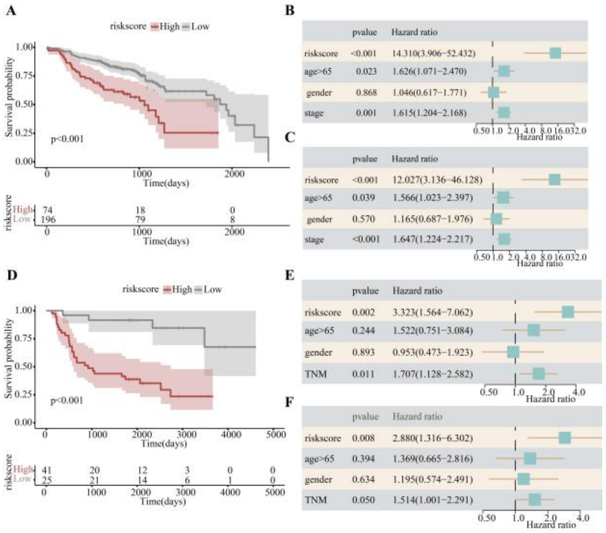

Figure S3. External validation of model genes: (A)The high-risk group in the external validation set of GSE65858 was significantly associated with poor survival prognosis.(B) Univariate regression analysis of risk scores in the external validation set of GSE65858. (C) Multivariate regression analysis of risk scores in the external validation set of GSE65858.(D) The high-risk group in the external validation set of GSE85446 was significantly associated with poor survival prognosis.(E) Univariate regression analysis of risk scores in the external validation set of GSE85446.(F) Multivariate regression analysis of risk scores in the external validation set of GSE85446.

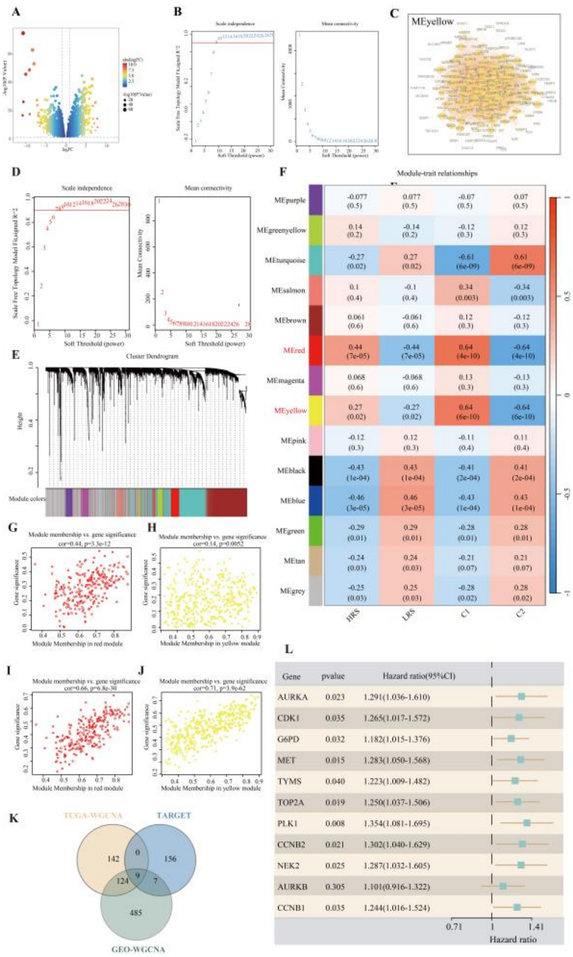

Figure S4.WGCNA analysis of machine learning model validation set GSE41613(A) TCGA-OSCC was screened by differential analysis ∣Log2FC∣ > 1 and P < 0.05 for differential genes included in WCGNA. Volcano plots were visualized for the differential genes. (B)A soft threshold of 9 was chosen for the module analysis. (C)Heatmap showing correlation between gene modules and selected phenotypes. (D)selection of soft threshold, the soft threshold was selected as 7 in GSE41613; (E)tree diagram of gene modules in GSE41613; (F)heat map of correlation between gene modules and OSCC Glycolysis High Risk Machine Learning Model and Glycolysis Adverse Prognosis subtypes in GSE41613; (G-H)scatter plot of red vs. Yellow module correlation analysis, the horizontal axis is the gene's intra-module connectivity (MM) in the current module, and the vertical axis is the gene's correlation coefficient (GS) with OSCC Glycolysis High Risk Machine Learning Model correlation coefficients (GS);(I-J)Red and yellow module correlation analysis scatter plots, horizontal axis is the intra-module connectivity (MM) of genes in the current module, vertical axis is the correlation coefficients (GS) of genes with poor prognostic subtypes of glycolysis;(K)Module genes were intersected with those of the AS/BJO-NEs anti-OSCC target, and the intersection targets are glycolysis-related genes (GRGs).(L)Univariate Cox analysis of GRGs and GHGs targeted by AS/BJO-NEs.

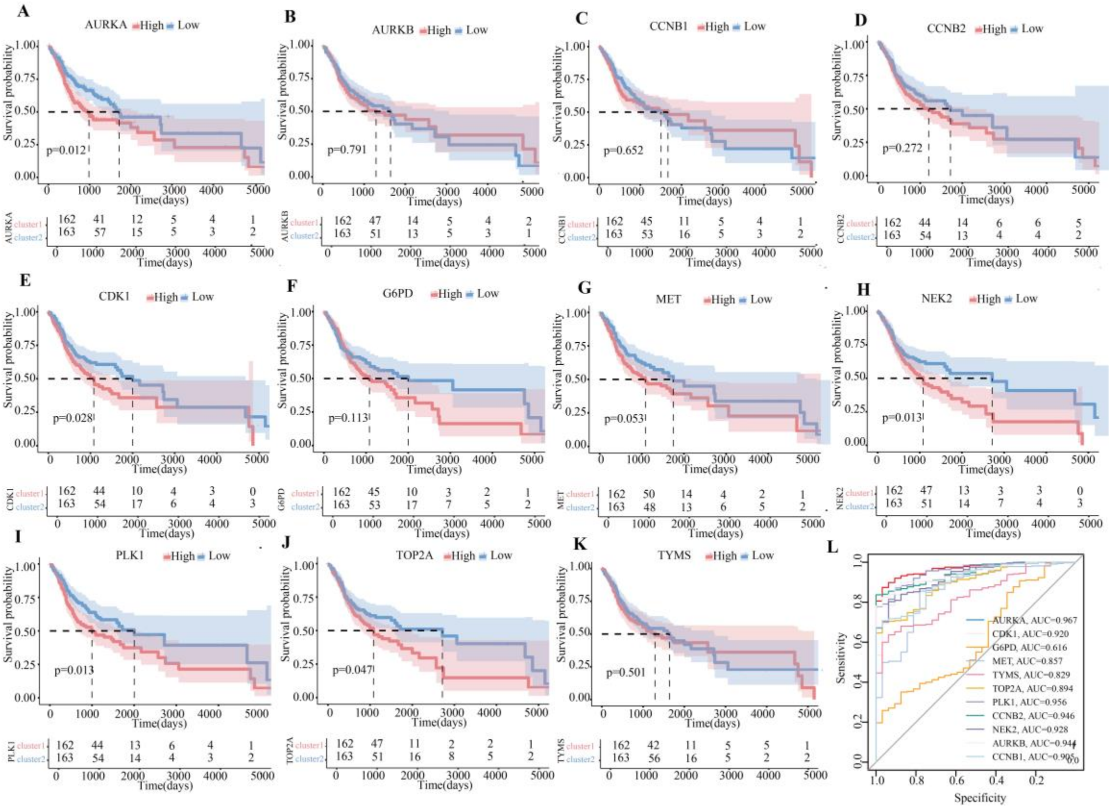

Figure S5. Survival analysis analysis and ROC analysis of the GHGs and GRGs targeted by AS/BJO-NEs: (A-K)Kaplan-Meier survival analysis of glycolysis hub and related genes.(L) ROC analysis of glycolysis hub and related genes.

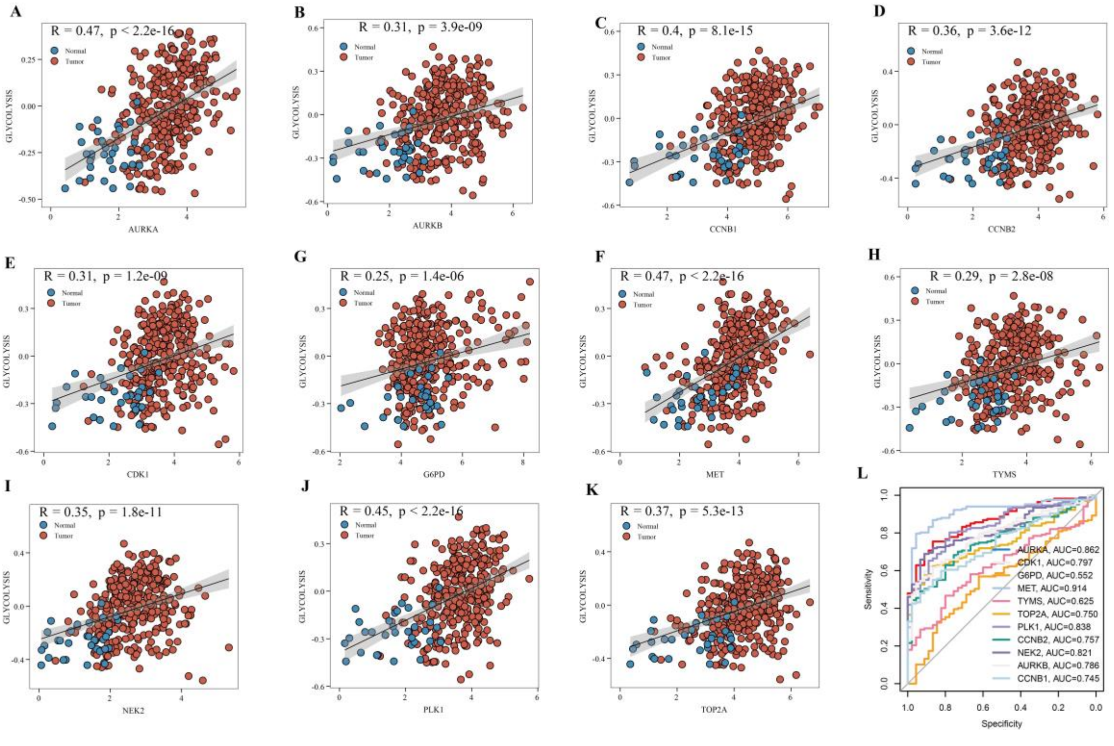

Figure S6:Correlation analysis and ROC analysis of the GHGs and GRGs targeted by AS/BJO-NEs with glycolytic metabolic levels:(A-K)Correlation analysis of AURKA, AURKB, CCNB1, CCNB2, CDK1, G6PD, MET, TYMS, NEK2, PLK1 and TOP2A with GSVA scores of glycolysis levels.(L)ROC analysis of

related genes.

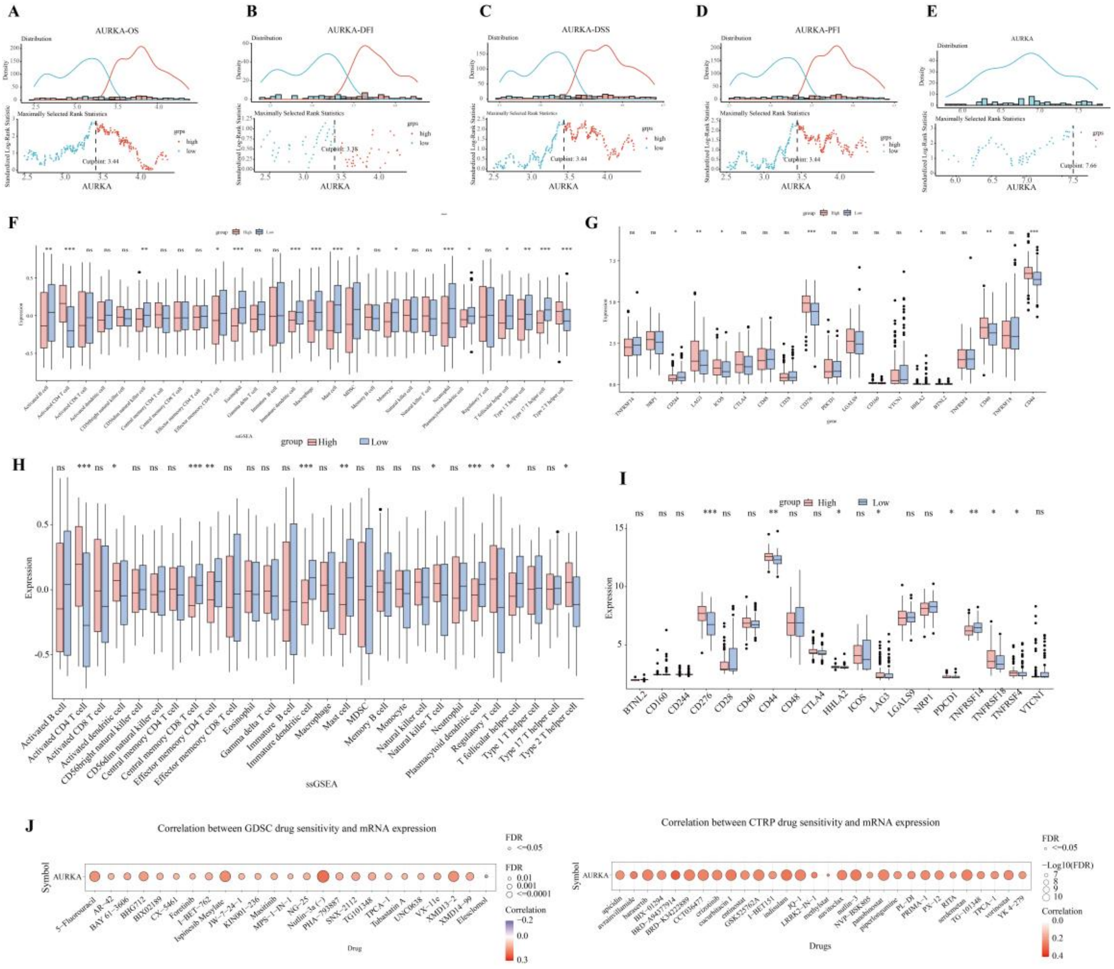

Figure S7:Optimal truncation and supplementary bioinformatics analysis of AURKA (A-D)Analysis of optimal cut-off values for AURKA expression based on TCGA-OSCC transcriptomic data and clinical survival data; (E)Analysis of optimal cut-off values for AURKA expression based on GSE41613 transcriptomic data and clinical survival data;(F-G) Immune cell infiltration (Figure F) and immune checkpoint expression (Figure G) were analyzed when AURKA was highly expressed in TCGA-OSCC;(H) Analysis of immune checkpoint expression based on GSE30784 grouped into median AURKA expression subgroups (AURKA high vs. low expression);(I)GSE30784 based immune infiltration analysis of ssGSEA grouped into median AURKA expression subgroups (AURKA high vs. low expression).(J) Drug sensitivity analysis of GSCA to AURKA.

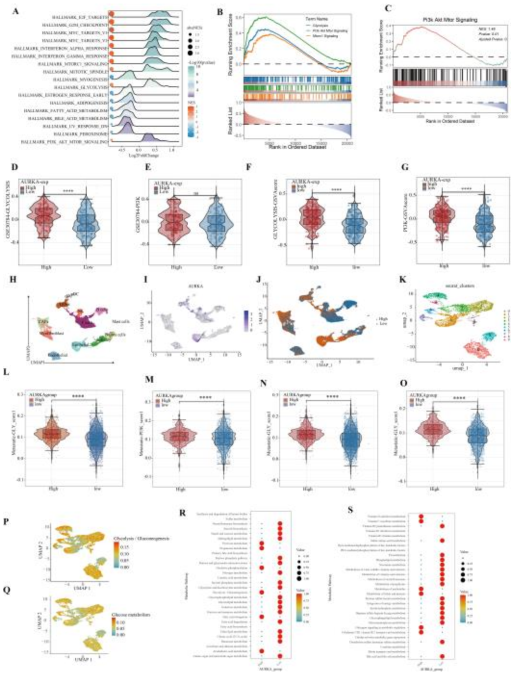

Figure S8.Supplementary validation of GSEA analysis:(A)GSEA analysis of AURKA single gene based on GSE30784 dataset;(B)AURKA was positively correlated with PI3K/AKT/MOTR pathway activity and glycolysis level in GSEA enrichment analysis;(C)AURKA expression was positively correlated with glycolysis level;(D-E)Analysis based on TCGA-OSCC transcriptome data: Differences in the level of glycolysis metabolism (Figure D) and PI3K/AKT pathway activation level (Figure E) between high/low expression of AURKA (grouped based on median expression, scored by GSVA algorithm);(F-G)Analysis based on TCGA-OSCC transcriptome data: Differences in the level of glycolysis metabolism (Figure F) and PI3K/AKT pathway activation level (Figure G) between high/low expression of AURKA (grouped based on median expression, scored by GSVA algorithm);(H).Single-cell datasets were categorized after downscaling and annotation into: T cells, B cells, NK, cDC, pDC, TAM, CAFs, Myofibroblast, Epithelial, Mast cells, etc.(I)AURKA is highly expressed mainly in epithelial cells.(J)AUCell assessed the level of glycolytic metabolism in each cell cluster, it was high in epithelial cells.(K)The epithelial cells were re-descended to form nine clusters.(L-M)AUCell evaluation showed that the level of glycolysis was higher in the AURKA high expression group in the epithelial cells of both primary (Figure L) and metastatic foci(Figure M).(N-O) AUCell analysis showed elevated PI3K/AKT pathway activation in epithelial cells with high AURKA expression, both in primary (Figure N) and metastatic lesions (Figure O). (P-Q) The level of glycolytic metabolism (Fig. P) and glucose uptake (Fig. Q) in the epithelium was assessed using “scMETABOLISM” and the results are shown in the “umap” diagram. (R-S) Using “scMETABOLISM" to assess the levels of different metabolic processes in KEGG (Fig. R) versus the REACTOME database (Fig. S) for epithelial cells. It was seen that glycolysis in KEGG was higher at high expression of AURKA, as well as glucose uptake in REACTOME, than at low expression.

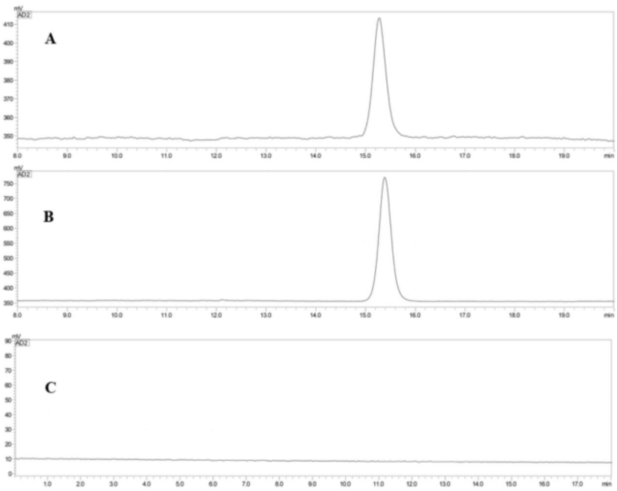

Figure S9: Specifiity examination of UPLC/MS spectra: (A) The chromatogram of AS/BJO-NEs (Test solution). (B) The chromatogram of AS-IV injection(Reference solution).(C) The chromatogram of the blank group (methanol and water solution). Test solution: Precisely measure 100 μL of the AS/BJO-NEs solution into a 1.5 mL ELP tube, add 400 μL of methanol, vortex for 5 minutes to break the emulsion, centrifuge at 12000 rpm for 10 minutes, and take the upper layer solution for testing. Reference solution preparation: Precisely weigh 3 mg of AS-IV in a 100 mL volumetric flask, add methanol for ultrasonic dissolution and make up to the mark, prepare a 30 μg/mL reference standard solution, store at 4℃. The blank group is the methanol and water solution.

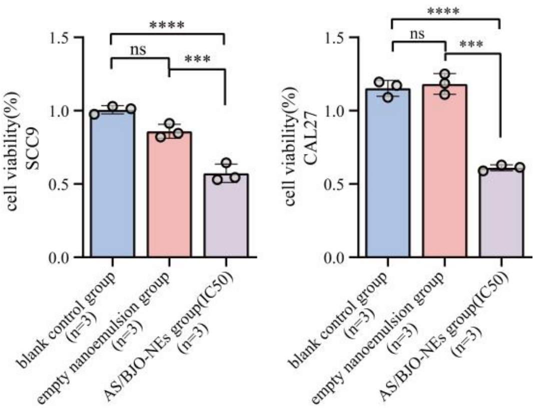

Figure S10:CCK8 supplementation experiment:The CCK8 assay ruled out the influence of the empty nanoemulsion.

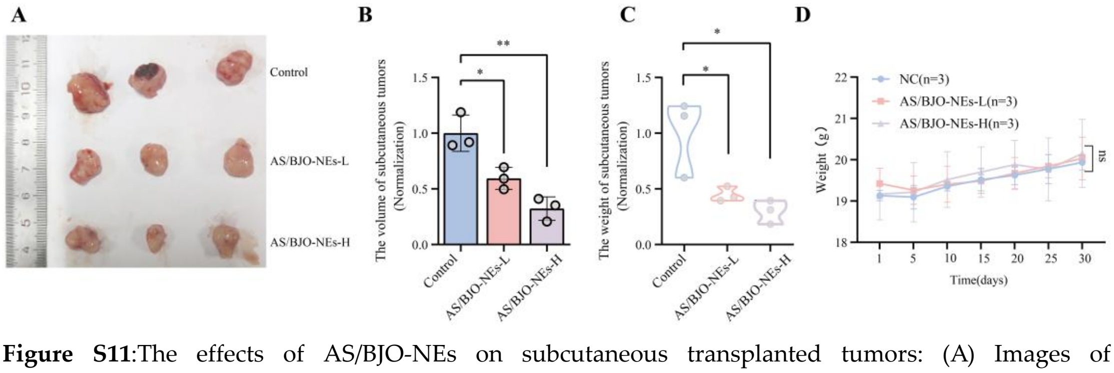

Figure S11:The effects of AS/BJO-NEs on subcutaneous transplanted tumors: (A) Images of subcutaneous tumors in each group; (B) The volume of subcutaneous tumors in the experimental group

was significantly different from that in the control group. (C) The mass of subcutaneous tumors in the experimental group was significantly different from that in the control group. (D)Quantitative graph of weight changes in each group of nude mice.*$p < 0.05$，**$p < 0.01$，***$p < 0.001$，****$p < 0.0001$。
