# Supplementary material for: Astragaloside–Brucea Javanica Oil Nanoemulsion Regulates Glycolysis in Oral Squamous Cell Carcinoma Through AURKA-Mediated PI3K/AKT/HIF-1α Pathway
Source: Pharmaceuticals (Basel). 2025 Nov 24;18(12):1783. doi: 10.3390/ph18121783 (PMC12736130; doi:10.3390/ph18121783)
Supplement: Supplementary file 1 [file pharmaceuticals-18-01783-s001.zip › Supplementary Table S11.pdf]

PCR section

<table>
<tr><th>Primer name</th><th>primer sequence</th><th>reaction system</th><th>PCR amplification conditions</th></tr>
<tr><td>AURKA -F</td><td>ATCGTGCAGGGGGAGAAATC</td><td rowspan="12">2X SYBR Green Pro Taq HS Premix：10μl;
Template*2：
2μl;
Primer F(10μM)：0.4μl
Primer R(10μM): 0.4μl;
ROX Reference Dye (4μM)*4, 5, 6: 0.4μl;
RNase free water: Up to 20μl;</td><td rowspan="12">95℃ 30s
95℃ 5s
(Forty cycles);
60℃ 30s</td></tr>
<tr><td>AURKA -R</td><td>GAGGGCAGCAGTCAATGGTA</td></tr>
<tr><td>AKT -F</td><td>GGACAAGGACGGGCACATTA</td></tr>
<tr><td>AKT -R</td><td>CGACCGCACATCATCTCGTA</td></tr>
<tr><td>PI3K-F</td><td>GACTGCCGAGAGATTTTCCCA</td></tr>
<tr><td>PI3K-R</td><td>CCAAATCTGAAGCAGCGCC</td></tr>
<tr><td>HIF1-a-F</td><td>TCTGCAACATGGAAGGTATTGC</td></tr>
<tr><td>HIF1-a-R</td><td>GCACCAAGCAGGTCATAGGT</td></tr>
<tr><td>PKM2-F</td><td>CCATGCGTGTTGTTCCTGTG</td></tr>
<tr><td>PKM2-R</td><td>TACAAGCGTTGCTGGCCTAA</td></tr>
<tr><td>HK2-F</td><td>GGCCACGATGTAGTCACCTT</td></tr>
<tr><td>HK2-R</td><td>CGGTCCCAACAATGAGTCCA</td></tr>
</table>
