# Supplementary material for: Astragaloside–Brucea Javanica Oil Nanoemulsion Regulates Glycolysis in Oral Squamous Cell Carcinoma Through AURKA-Mediated PI3K/AKT/HIF-1α Pathway
Source: Pharmaceuticals (Basel). 2025 Nov 24;18(12):1783. doi: 10.3390/ph18121783 (PMC12736130; doi:10.3390/ph18121783)
Supplement: Supplementary file 1 [file pharmaceuticals-18-01783-s001.zip › Supplementary Table S7.pdf]

Group	Inhibition ratio(%)
Experimental Group 1	86
Experimental Group 2	75
Experimental Group 3	80
Experimental Group 4	72
Experimental Group 5	78
Experimental Group 6	81
Experimental Group 7	80
Experimental Group 8	81
Experimental Group 9	82
Experimental Group 10	82
Experimental Group 11	83
Experimental Group 12	68
Experimental Group 13	73
Control Group 1	52
Control Group 3	55
Control Group 3	7

Experimental Group 1, which contained 1.40 g glycerol, 0.79 g soybean phospholipid, 0.6 ml BJO, and 0.06 g AS-IV and was processed at 538 bar homogenization pressure.

Experimental Group 2: The prescription provided by Experimental Group 2 is roughly the same as that provided by Experimental Group 1, with the exception that the BJO in Experimental Group 2 is 0.3 ml.

Experimental Group 3: The prescription provided by Experimental Group 3 is roughly the same as that provided by Experimental Group 1, with the difference being that the BJO in Experimental Group 3 is 0.8 ml.

Experimental Group 4: The prescription provided by Experimental Group 4 is roughly the same as that provided by Experimental Group 1, with the exception that the AS-IV in Experimental Group 4 is 0.03 g.

Experimental Group 5: The prescription provided by Experimental Group 5 is roughly the same as that provided by Experimental Group 1, with the difference being that the AS-IV in Experimental Group 5 is 0.08 g.

Experimental Group 6: The prescription provided by Experimental Group 6 is roughly the same as that provided by Experimental Group 1, with the exception that the soybean oil in Experimental Group 6 is 5 g.

Experimental Group 7: The prescription provided by Experimental Group 7 is roughly the same as that provided by Experimental Group 1, with the difference being that the soybean oil in Experimental Group 7 is 15 g.

Experimental Group 8: The prescription provided by Experimental Group 8 is roughly the same as that provided by Experimental Group 1, with the exception that the soybean phospholipids in Experimental Group 8 are 0.5 g.

Experimental Group 9: The prescription provided by Experimental Group 9 is roughly the same as that provided by Experimental Group 1, with the difference being that the soybean phospholipids in Experimental Group 9 are 1 g.
Experimental Group 10: The prescription provided by Experimental Group 10 is roughly the same as that provided by Experimental Group 1, with the exception that the glycerol in Experimental Group 10 is 0.8 g.
Experimental Group 11: The prescription provided by Experimental Group 11 is roughly the same as that provided by Experimental Group 1, with the difference being that the glycerol in Experimental Group 11 is 2 g.
Experimental Group 12: The prescription provided by Experimental Group 12 is roughly the same as that provided by Experimental Group 1, with the exception that the BJO in Experimental Group 12 is 0.3 ml and the AS-IV is 0.03 g.
Experimental Group 13: The prescription provided by Experimental Group 13 is roughly the same as that provided by Experimental Group 1, with the difference being that the BJO in Experimental Group 13 is 0.6 ml and the AS-IV is 0.06 g.
Control Group 1: The prescription provided by Control Group 1 is roughly the same as that provided by Experimental Group 1, with the exception that there is no BJO in Control Group 1.
Control Group 2: The prescription provided by Control Group 2 is roughly the same as that provided by Experimental Group 1, with the exception that there is no AS-IV in Control Group 2.
Control Group 3: The prescription provided by Control Group 3 is roughly the same as that provided by Experimental Group 1, with the difference being that there is neither AS-IV nor BJO in Control Group 3.
Experiment process: Specifically, the prescriptions of the above experimental groups and control groups were respectively prepared into corresponding finished products, and then diluted 100 times before being applied to CAL27 cells for a cell survival inhibition test.
